# All Roads Lead to Carbinolamine: QM/MM Study of Enzymatic C–N Bond Cleavage in Anaerobic Glycyl Radical Enzyme Choline Trimethylamine-Lyase (CutC)

**DOI:** 10.1021/acs.jpcb.5c04023

**Published:** 2025-09-08

**Authors:** Marko Hanzevacki, J. Jasmin Güven, Philip Hinchliffe, John Shaw, Antonia S. J. S. Mey, Natalie Fey, James Spencer, Adrian J. Mulholland

**Affiliations:** † Centre for Computational Chemistry, School of Chemistry, 1980 University of Bristol, Bristol BS8 1TS, U.K.; ‡ EaStCHEM School of Chemistry, 3124 University of Edinburgh, Edinburgh EH9 3FJ, U.K.; § School of Cellular and Molecular Medicine, University of Bristol, Bristol BS8 1TD, U.K.

## Abstract

The anaerobic glycyl radical enzyme choline trimethylamine-lyase (CutC) is produced by multiple bacterial species in the human gut microbiome and catalyzes the conversion of choline to trimethylamine (TMA) and acetaldehyde. CutC has emerged as a promising therapeutic target due to its role in producing TMA, which is subsequently oxidized in the liver to form trimethylamine-*N*-oxide (TMAO). Elevated TMAO levels are associated with several human diseases, including atherosclerosis and other cardiovascular disorders—a leading cause of mortality worldwide. Understanding the catalytic mechanism of this enzyme should aid successful design of potent inhibitors. Here, we employed extensive molecular dynamics (MD) simulations to reveal that hydrogen bonding within the CutC active site plays a crucial role in orienting choline for the initial *pro*-S hydrogen abstraction, leading to the formation of the α-hydroxy radical. The reaction mechanism was explored with quantum mechanics/molecular mechanics (QM/MM). The performance of three density functionals (B3LYP-D3, ωB97X-D3, and M06–2X) was tested against DLPNO–CCSD­(T) ab initio calculations. These results indicate that choline cleavage occurs via TMA migration leading to a stable product carbinolamine which likely undergoes 1,2-elimination to acetaldehyde and TMA in water. Mechanistic insights consistently support the TMA migration pathway over direct TMA elimination, providing clear evidence for the preferred reaction mechanism. Two distinct mechanistic pathways were identified: one with a relatively high activation energy barrier, and the other with a lower barrier which is in a good agreement with the previously reported experimental kinetic parameters. QM/MM MD simulations further confirm that Glu491 functions as a catalytic base, abstracting a proton from the α-hydroxy radical and thereby facilitating the experimentally observed C–N bond cleavage. The relative binding affinity of the reactant (choline) and product (carbinolamine) was estimated with alchemical relative binding free energy calculations, complemented by noncovalent interaction analysis. These results elucidate the molecular basis for differences in their interactions with CutC (particularly highlighting key electrostatic interactions with Asp216 and Glu491) providing insights for future inhibitor design.

## Graphical abstract

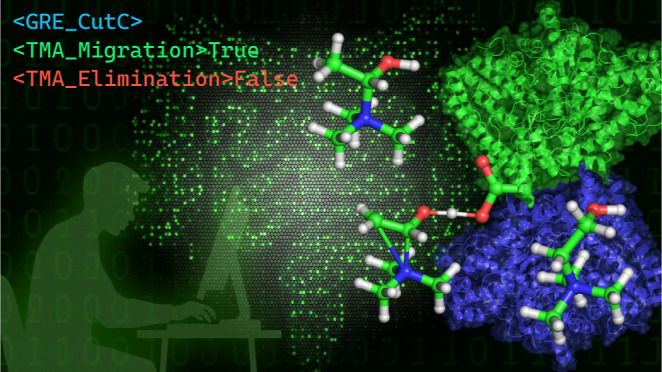

## Introduction

Choline trimethylamine-lyase (CutC) is a glycyl radical enzyme (GRE) that plays a pivotal role in microbial metabolism, particularly in the anaerobic degradation of the essential nutrient choline.^1–3^ Found predominantly in the anaerobic gut microbiota, CutC catalyzes the cleavage of choline into trimethylamine (TMA) and acetaldehyde through a radical-mediated mechanism (Figure 1a).^4^ This enzymatic reaction has significant implications for human health, as TMA is further oxidized in the liver to form trimethylamine-*N*-oxide (TMAO), a metabolite strongly associated with colorectal cancer, nonalcoholic fatty liver disease, cardiovascular disorders, atherosclerosis, chronic kidney disease, type 2 diabetes, and other metabolic disorders such as trimethylaminuria, also known as fish odor syndrome.^5–7^ Understanding the mechanism of CutC should help elucidate the biochemical basis of TMAO-related pathologies and for developing potential therapeutic interventions targeting this metabolic pathway.^8–10^

**Figure 1:**
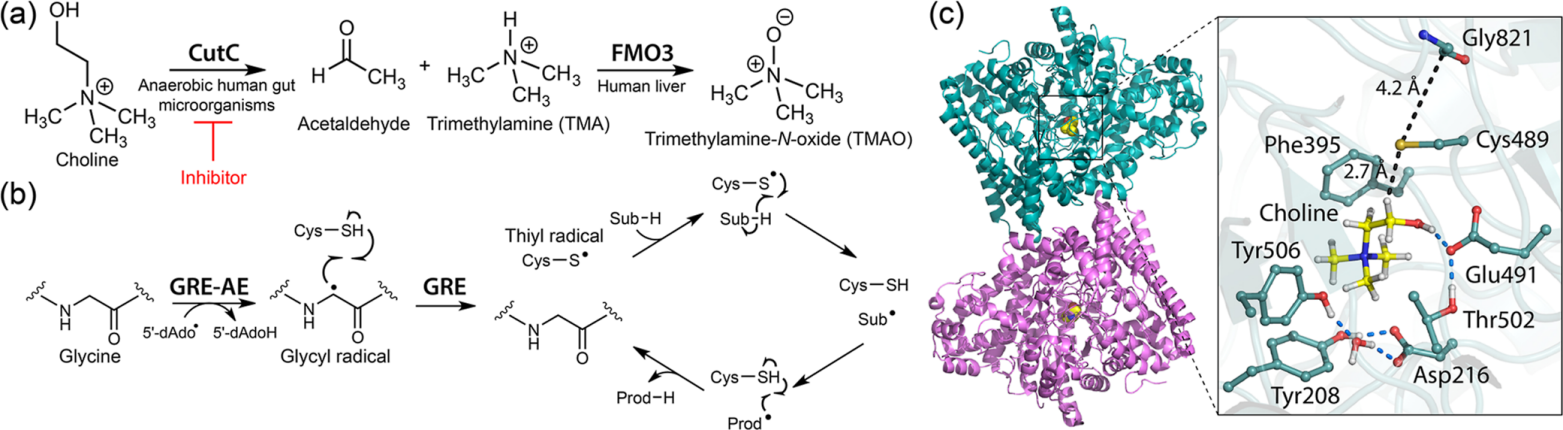
CutC reaction and activation. (a) CutC catalyzes the degradation of choline to trimethylamine (TMA) and acetaldehyde; TMA is subsequently oxidized by flavin-containing monooxygenase 3 (FMO3) to form the disease-associated metabolite trimethylamine-*N*-oxide (TMAO). (b) Mechanism of glycyl radical formation in glycyl radical enzymes (GREs): The GRE-activating enzyme transfers an electron from an iron–sulfur cluster to *S*-adenosylmethionine (SAM), leading to reductive cleavage of SAM into methionine and a 5′-deoxyadenosyl radical (5′-dAdo^•^). This radical abstracts a hydrogen atom from the active-site glycine, generating the catalytically essential glycyl radical. Subsequent hydrogen transfer between the glycyl radical and a nearby cysteine residue forms a thiyl radical, which initiates substrate C–H bond cleavage during the GRE catalytic cycle. (c) Crystal structure of CutC in complex with choline (PDB ID 5FAU), illustrating the substrate-binding site and key catalytic residues.

CutC belongs to the GRE superfamily, a class of enzymes that utilize a glycyl radical cofactor for catalysis.^11–13^ The activation of CutC requires an activating enzyme, CutD, which uses *S*-adenosylmethionine (SAM) and an iron–sulfur cluster to generate the glycyl radical on a conserved glycine residue of CutC.^14^ This radical is essential for initiating the catalytic cleavage of choline. The glycyl radical then abstracts a hydrogen atom from a catalytic cysteine residue, generating a thiyl radical that serves as the proximal initiator of C–N bond cleavage (Figure 1b).

Despite its biological importance, the precise molecular mechanism governing CutC activity remains incompletely understood. Experimental investigations, particularly X-ray crystallography, have provided understanding of the structural and functional properties of CutC.^3,8,15,16^ For example, the crystal structure of homodimer CutC in complex with choline identified interactions between the substrate and catalytic residues, and suggested possible mechanisms of turnover (Figure 1c).^15^

The postulated mechanism (Figure 2), based on crystallographic data and site-directed mutagenesis studies, involves initial hydrogen transfer from choline (specie A, Figure 2) by the Cys489 thiyl radical forming an α-hydroxy radical (specie B) followed by deprotonation of the choline hydroxy group by Glu491.^3,15^ This triggers a lone pair movement coupled with a spin-center shift, facilitating C–N bond cleavage (specie C). It is unknown whether the mechanism involves direct elimination of TMA (species D and E), or it rather proceeds through migration of the trimethylammonium moiety to generate a stable hemiaminal (carbinolamine, species F and G).^11,17^ Another poorly understood step in the mechanism is the deprotonation of Glu491 at the end of the catalytic cycle. It has been debated whether direct deprotonation by TMA is feasible, or whether it instead occurs via a proton transfer network involving Asp216 and Thr502, which may adopt multiple conformations to act as proton donor and acceptor (specie D).^17,18^

**Figure 2:**
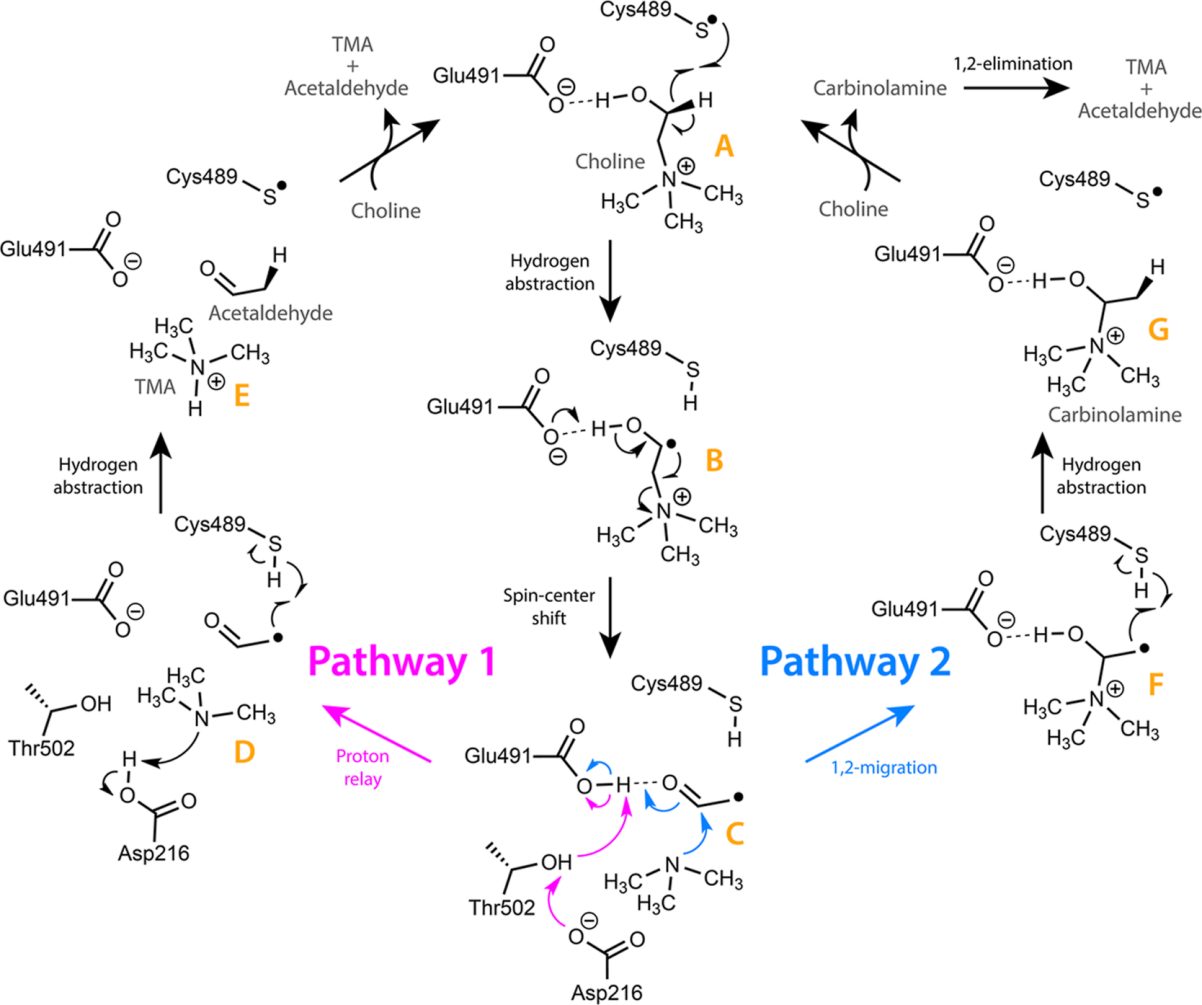
Proposed mechanism of choline cleavage by CutC. The reaction begins with a thiyl radical abstracting a hydrogen atom from the C1 position of choline, forming an α-hydroxyalkyl radical intermediate. This radical can then follow two possible pathways to generate trimethylamine (TMA) and acetaldehyde in the CutC active site. In Pathway 1, the α-hydroxyalkyl radical undergoes base-catalyzed direct elimination of TMA via a spin-center shift, with protonation of the departing TMA possibly involving a proton relay and rearrangement of active site residues. In Pathway 2, there is a 1,2-migration of the trimethylammonium group, producing a carbinolamine intermediate that spontaneously decomposes (1,2-elimination) to release TMA and acetaldehyde. The experimentally determined turnover number (*k*_cat_) of 157 ± 2 s^–1^ corresponds to an activation free energy (Δ*G* ^⧧^) of 14.6 kcal mol^–1^ at 300 K.

In this work, we combine multiscale computational approaches with prior experimental data to provide a comprehensive analysis of the catalytic mechanism of CutC and its structure–function relationships. By employing molecular dynamics (MD) simulations, hybrid quantum mechanics/molecular mechanics (QM/MM) calculations, and relative binding free energy analyses, we aim to resolve questions regarding the chemical steps involved in choline cleavage and the roles of specific active site residues. This integrated approach not only provides detailed mechanistic insight into choline cleavage by CutC, but also identifies key molecular interactions that are critical for substrate and product binding. Together, these findings establish a foundation for the rational development of selective CutC inhibitors and new strategies for modulating microbial choline metabolism.

## Methods

### System Preparation

Coordinates for wild-type CutC from *D. alaskensis* bound to choline (PDB ID 5FAU, chain A) were used.^15^ Protonation states of titratable residues were assigned using the H++ server http://biophysics.cs.vt.edu/H++.^19^ All crystal water molecules were retained, and the cocrystallized glycerol was removed. Standard amino acids were modeled with the ff14SB force field;^20^ nonstandard residues (thiyl radical and choline) were parametrized using RESP charges at the HF/6-31G­(d)//B3LYP/6-31G­(d) level and GAFF atom types (see Data and Software Availability for parameters).^21–24^ All geometries and ESP charges were obtained with Gaussian16.^25^ The enzyme–substrate complex was solvated in a cubic TIP3P^26^ water box and neutralized with Na^+^ ions using the AMBER24 suite.^27,28^

### Molecular Dynamics Simulations

The solvated CutC system underwent restrained energy minimization (10,000 cycles; first 200 steepest descent, remainder conjugate gradient) with positional restraints (10 kcal mol^–1^ Å^–2^) on backbone heavy atoms. This was followed by gradual heating from 100 to 300 K over 2 ns (*NVT* ensemble, Langevin thermostat), and 2 ns equilibration at 300 K and 1 atm (*NPT* ensemble, Monte Carlo barostat performing volume change attempts every 100 steps), both with backbone restraints. An additional 2 ns unrestrained equilibration was performed. Production MD simulations were conducted in the *NPT* ensemble at 300 K and 1 atm for 200 ns per replica (three replicas per system, total 600 ns), saving trajectory frames every 20 ps. All simulations employed *pmemd.cuda* in AMBER24,^27^ with SHAKE^29^ constraints on bonds to hydrogen, PME for long-range electrostatics (8.0 Å cutoff), and a 2 fs time step.

### QM/MM Calculations

Representative snapshots for QM/MM calculations were extracted from MM MD trajectories by monitoring key interatomic distances critical to catalysis. Specifically, frames were selected where the distance between the sulfur atom of Cys489 and the *pro-S* hydrogen atom of choline was approximately 3 Å, and the distance between the hydroxyl proton of choline and the OE2 atom of Glu491 was near 1.7 Å, corresponding to configurations poised for hydrogen atom and proton transfer events essential to the reaction mechanism. A representative MD snapshot of the CutC–choline complex was processed with *cpptraj* of AMBER24 ^27^ to generate a nonperiodic, truncated system. The closest 500 water molecules to the QM region were retained to form the solvation shell. The active region, defined as all residues with atoms within 8.0 Å of the QM region, was allowed to move during geometry optimization; all other atoms were fixed. To systematically evaluate the influence of QM region size, we defined four QM regions of increasing complexity as shown in Figure S1. Minimal QM region includes the substrate choline, Cys489, and Glu491. Medium QM region expands the minimal region by adding Asp216 and Thr502. Large QM region further includes Tyr208 and Phe395. Extra-large QM region incorporates an additional active site water molecule along with His209, Thr334, Phe389, Met487, Tyr506, Leu698, and Ile700. The total charge of the QM region varied between 0 and −1 depending on the residues included, while the spin multiplicity was always 2. MM atoms directly bonded to QM atoms were capped with link hydrogens and treated using covalent coupling. The charge shift scheme was applied to mitigate overpolarization of the QM region by MM centers.^30^ The cutoff for QM/MM electrostatic interactions was set to 99.0 Å. Geometry optimizations were performed using electrostatic embedding and the additive scheme at the UB3LYP/6-31G­(d):AMBER QM/MM level, including Grimme’s D3 dispersion corrections.^31,32^ The L-BFGS algorithm was used for minimization.^33,34^ Transition state searches were conducted using the climbing image nudged elastic band (CI-NEB) method,^35^ followed by refinement with the dimer method. Frequency calculations at the optimization level confirmed the nature of the stationary points. To benchmark the performance of different density functionals, QM/MM single-point energy calculations were performed on the optimized geometries using B3LYP-D3,^31,36^ ωB97X-D3,^37^ and M06-2X^38^ functionals, all with the def2-TZVP basis set. Results were compared to calculations with the DLPNO–CCSD­(T) method,^39^ which employed the cc-pVQZ/C basis set, performing a three-point extrapolation to approach the complete basis set limit. All QM/MM calculations were carried out using the ORCA 5.0.4 ^40^/DL_POLY_5 ^41^ interface within Py-ChemShell.^42^

### QM/MM Molecular Dynamics Simulations

While high-level ab initio QM/MM single-point calculations yield accurate reaction energetics, direct sampling of enzyme conformational dynamics via full QM/MM MD umbrella sampling simulations at this level is prohibitive for systems of our size due to excessive computational cost. In this work, we performed extensive sampling using QM/MM molecular dynamics simulations to explicitly account for enzyme flexibility during the reaction. By employing QM/MM MD umbrella sampling at the DFT level, we effectively probe the dynamic conformational changes associated with catalysis. Although computationally demanding, these simulations offer a balance between conformational sampling and accuracy. The QM/MM MD simulations were carried to explore the dynamical coupling between the proton transfer and the C–N bond cleavage. All QM/MM MD simulations were performed with the *sander* module in AMBER24.^27^ Systems were first energy-minimized (20 steps steepest descent, 80 steps conjugate gradient), followed by a 1 ps equilibration at 300 K using Langevin dynamics. Production QM/MM MD simulations were conducted for 50 ps with a 1 fs time step, starting from pre-equilibrated structures of three CutC states: choline, α-hydroxy radical, and carbinolamine obtained from umbrella sampling QM/MM MD simulations. It is important to emphasize that QM/MM MD simulations at the DFT level are computationally demanding, and simulation times on the order of tens of picoseconds are consistent with time scales commonly reported in comparable enzyme mechanistic studies.^18^ Such durations are sufficient to capture multiple spontaneous occurrences of key coupled events, notably proton transfer and C–N bond cleavage, which are central to the CutC reaction mechanism. By performing simulations that adequately sample these critical reaction steps, we ensure reliable statistical representation to construct free energy landscapes from the observed event probabilities. Additionally, umbrella sampling was utilized to enhance sampling efficiency along relevant reaction coordinates, thereby strengthening the robustness of the mechanistic insights derived from these computationally demanding simulations.

QM/MM MD umbrella sampling simulations were performed to obtain representative structures of the α-hydroxy radical and carbinolamine intermediates formed from choline. The reaction coordinate for the first hydrogen transfer was defined as a linear combination of the Cα–Hα and S_Cys_–Hα bond distances, describing the hydrogen abstraction from choline by the Cys489 thiyl radical, with values ranging from −1.5 Å to 0.9 Å. For the second hydrogen transfer, the reaction coordinate was defined similarly as a linear combination of the S_Cys_–Hα and Cβ–Hα distances (with values ranging from −1.4 Å to 1.5 Å), representing hydrogen abstraction from Cys489 by the vinoxy radical. Each coordinate was sampled in 0.1 Å increments using harmonic restraints with a force constant of 200 kcal mol^–1^ Å^–2^. For each umbrella window, the system was energy-minimized for 100 steps (20 steepest descent followed by 80 conjugate gradient), equilibrated for 1 ps, and then subjected to 4 ps of production MD (total of 100–120 ps of sampling) at 300 K using Langevin dynamics. The potential of mean force (PMF) profiles were constructed using the weighted histogram analysis method (WHAM).^43,44^

Due to the substantial computational demands of QM/MM MD simulations combined with enhanced sampling, the medium QM region was chosen (Figure S1). This region includes key catalytic residues and provides accuracy comparable to larger QM regions, while capturing essential chemical steps—hydrogen atom transfer, proton transfer, and C–N bond cleavage—without compromising computational feasibility. The QM region was modeled at the UB3LYP/6-31G­(d) level (including D3 corrections with Becke–Johnson (BJ) damping)^45^ using the GPU-accelerated QUICK program interfaced with AMBER24.^27,46–48^ The QM region was assigned a total charge of −1 and a spin multiplicity of 2; no constraints were applied to hydrogen atoms within the QM region. QM/MM coupling employed electrostatic embedding, with direct-space electrostatic interactions included within an 8.0 Å cutoff.

### Alchemical Relative Binding Free Energy Simulations

The relative binding free energy (RBFE) between choline and carbinolamine was computed using alchemical free energy calculations in BioSimSpace.^49^ Forward (choline → carbinolamine) and backward (carbinolamine → choline) transformations were mapped using Lead Optimization Mapper (LOMAP),^50^ aligning the TMA groups to minimize perturbation. Systems were prepared in both unbound (water only) and bound (CutC with cysteine or thiyl radical, in water) states, solvated with TIP3P water^26^ using the *leap* module of AMBER24.^27^ Both states were minimized and equilibrated via NVT and NPT MD simulations in AMBER24 with BioSimSpace.^27,49^ For each transformation, three repeats were simulated, each using 15 λ-windows sampled for 5 ns with the SOMD engine (Sire).^51^ The phase space overlap and free energy convergence were assessed with alchemlyb^52^ in BioSimSpace (see Supporting Information for details).

### Analysis

Electronic structures of possible intermediates formed after the initial hydrogen atom abstraction and subsequent deprotonation of choline were computed at the B3LYP-D3BJ/def2-SVP level of theory in the gas phase and with CPCM implicit solvation (ε = 4)^53,54^ using the ORCA 6.0 program.^55^ Molecular visualization was carried out using PyMOL 3.1 ^56^ and VMD 1.9.3.^57^ Noncovalent interaction (NCI) analysis based on the independent gradient model^58^ was carried out in Multiwfn^59^ using representative snapshots from QM/MM MD simulations.^60^ The free energy landscape (FEL) was analyzed using a tool available at GitHub https://github.com/sulfierry/free_energy_landscape.git. Binding free energies were estimated using the MM/GBSA approach implemented in the MMPBSA.py module of AmberTools.^61,62^ The analysis employed 150 evenly spaced snapshots extracted from MM MD trajectories of CutC complexed with choline and carbinolamine. Solvation free energies were computed using the generalized Born implicit solvent model with the OBC2 parameter set,^63^ and mbondi2 atomic radii, at a salt concentration of 0.15 M. Nonpolar solvation contributions were estimated via solvent-accessible surface areas calculated by the LCPO method.^64^ For each snapshot, the free energies of the complex, receptor, and ligand were calculated, and the binding free energy was determined as the difference between these states. Additionally, per-residue energy decomposition analysis was performed to determine electrostatic, van der Waals, polar and nonpolar solvation contributions of individual residues to the overall binding free energy.

## Results and Discussion

### Hydrogen Bonding is important for *pro-S* H-Abstraction Forming an α-hydroxy Radical

Extensive MM MD simulations explored the conformational free energy landscape (FEL) of the choline substate in the active site of CutC (Figure 3). The FEL was visualized using two important distances, which indicated the reactivity of choline toward hydrogen abstraction by the thiyl radical (H_Choline_–SG_Cys_) and deprotonation by the glutamate base (HO_Choline_–OE2_Glu_).

**Figure 3:**
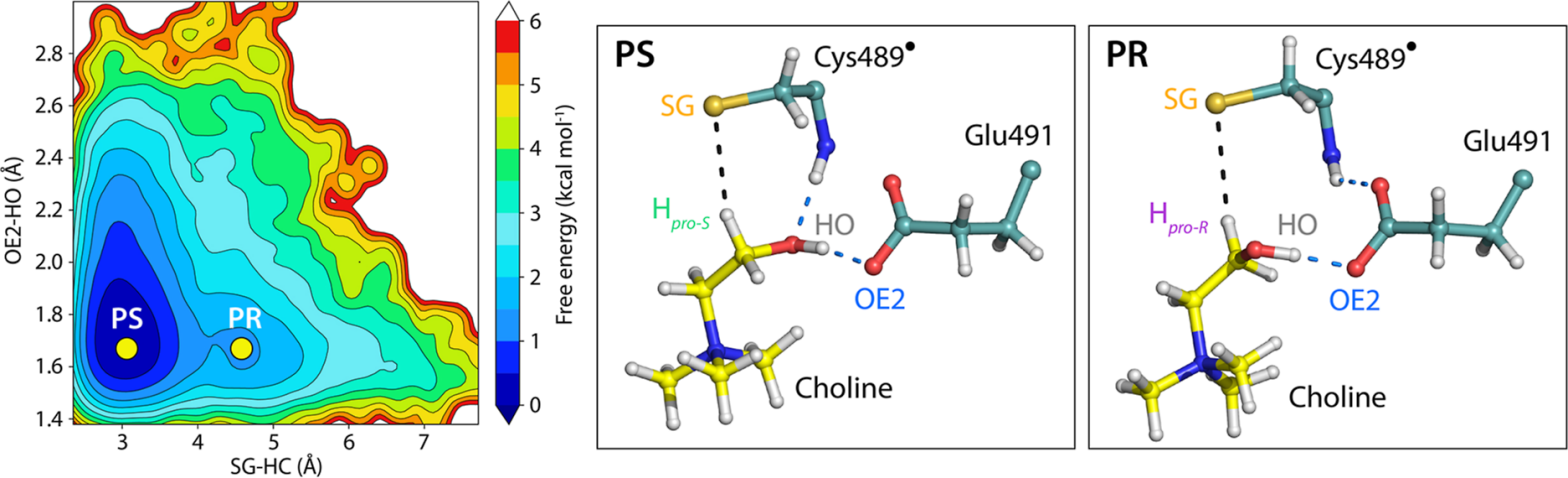
Free energy landscape (FEL) of choline binding in CutC, calculated from molecular mechanics molecular dynamics (MM MD) simulations at 300 K. The FEL reveals two distinct choline conformations, PS and PR, within the active site (for clarity only the choline, Cys489 and Glu491 are shown). The landscape is constructed using two collective variables: the distance between the *pro-S* hydrogen of choline and the sulfur atom of Cys489, and the distance between the choline hydroxy proton and the OE2 atom of Glu491, capturing both the hydrogen abstraction and proton transfer steps.

Two distinct conformations of choline were observed. The dominant conformation PS (*pro-S*) was more than 1 kcal mol^–1^ lower in energy compared to conformation PR (*pro-R*). A similar distribution of conformers was observed by calculating the choline OH–Cα–Cβ–N dihedral, with the average value of the PS dihedral closely resembling the conformation found in the crystal structure (Figure S2). The greater stability of PS can be explained by the additional hydrogen bond formed between the oxygen from the choline hydroxy group and the backbone NH of Cys489, which was absent in conformation PR. In both conformations, the hydroxy group of choline frequently formed a hydrogen bond with the OE2 of the Glu491 carboxylate side chain while OE1 was involved in a hydrogen bond with the backbone amino group of Val490 (see structures in Figure 3).

The hydrogen bonding between the OH group of choline and active site residues plays an important role in the stereoselectivity of the CutC thiyl radical toward extracting the *pro-S* hydrogen from the α-position relative to the OH group. This hydrogen is located around 3 Å from the SG of Cys489, positioning it for abstraction in the initial step of the reaction. Furthermore, this binding pose of choline in the active site probably prevents the β-hydrogen abstraction and the formation of unstable β-hydroxy radical. The presence of the negatively charged Asp216 and Glu491 residues probably contributes to additional stabilization of positively charged choline via electrostatic interactions.

### Electronic Structure Calculations Provide Insights into the Stability of Radical Intermediates

QM calculations investigated the intrinsic stability of radical intermediates potentially formed during choline degradation. These calculations were conducted on isolated molecules in vacuo (Figure S3), thus excluding the influence of the enzyme environment. The primary aim was to elucidate the general stability trends of the α- and β-hydroxy radical intermediates generated following the initial hydrogen abstraction from choline.

The choline hydroxy group has a relatively low *pK*_a_ ∼ 13.9, which is notably more acidic than that of a typical alcohol.^15^ This increased acidity favors deprotonation. Furthermore, it is well established that the acidity of the OH group in α-hydroxy radicals is further enhanced (typically lowered by an additional 4–8 pH units) making deprotonation even more favorable.^15^

The QM calculations indicate that deprotonation of the OH group in the α-hydroxy radical intermediate promotes the experimentally observed heterolytic cleavage of the C–N bond. This is shown by favorable spin density delocalization and by the antiperiplanar orientation between the C–N antibonding σ* orbital and the p-orbital of the carbon-centered radical, which facilitates hyperconjugative interactions.^15^ In contrast, deprotonation of the β-hydroxy radical intermediate leads to the formation of a highly unstable species. In this case, the spin density is more localized on the β-carbon atom relative to the OH group, and there is a lack of resonance stabilization. Additionally, the proximity of the radical center to the adjacent quaternary ammonium nitrogen atom results in significant charge repulsion, further destabilizing the β-hydroxy radical.

The formation of the β-hydroxy radical is probably further disfavored in the enzyme due to the binding mode of choline within the active site. This orientation positions the relevant hydrogen atoms far from the sulfur atom of the thiyl radical, impeding efficient hydrogen abstraction (see Figure 3).

### Static QM/MM Calculations Provide Evidence for a 1,2-Migration-Based Mechanism in CutC-Catalyzed Choline Cleavage

The single-conformation static QM/MM investigation elucidates the mechanistic intricacies of choline cleavage by CutC, revealing a finely tuned interplay between hydrogen/proton transfer and a crucial 1,2-migration of the TMA group to carbinolamine.

Notably, our calculations rule out an alternative, literature-proposed mechanism involving direct elimination of TMA via a proton relay (Glu491, Thr502, Asp216) (Figure 2a).^11,15,17^ Almost all optimized intermediates along this route (obtained with medium QM region) had significantly higher energy (Δ*E* = 4–10 kcal mol^–1^) than the highest energy intermediates of the migration pathways, rendering this mechanism unlikely (see Figure S4 for structures and energies). This reinforces the conclusion that CutC catalyzes C–N bond cleavage in choline through a 1,2-migration of the TMA group, rather than a direct elimination.

Two distinct mechanistic pathways were characterized (see QM/MM potential energy profiles including zero-point energy corrections in Figure 4), both converging on carbinolamine formation via a sequence of hydrogen atom transfers (HATs) and proton transfers, consistent with the mechanism involving TMA migration (see Figure 2b). According to DLPNO–CCSD­(T) energies, the first pathway (direct HAT from Cys489 to vinoxy radical) has an activation barrier of 19.6 kcal mol^–1^, while the second pathway (stepwise TMA migration followed by HAT) is substantially more favorable, with a barrier of 13.5 kcal mol^–1^, closely matching the experimentally determined activation energy of 14.6 kcal mol^–1^.^15^ This 6.1 kcal mol^–1^ difference favors the second stepwise pathway as the physiologically relevant route.

**Figure 4:**
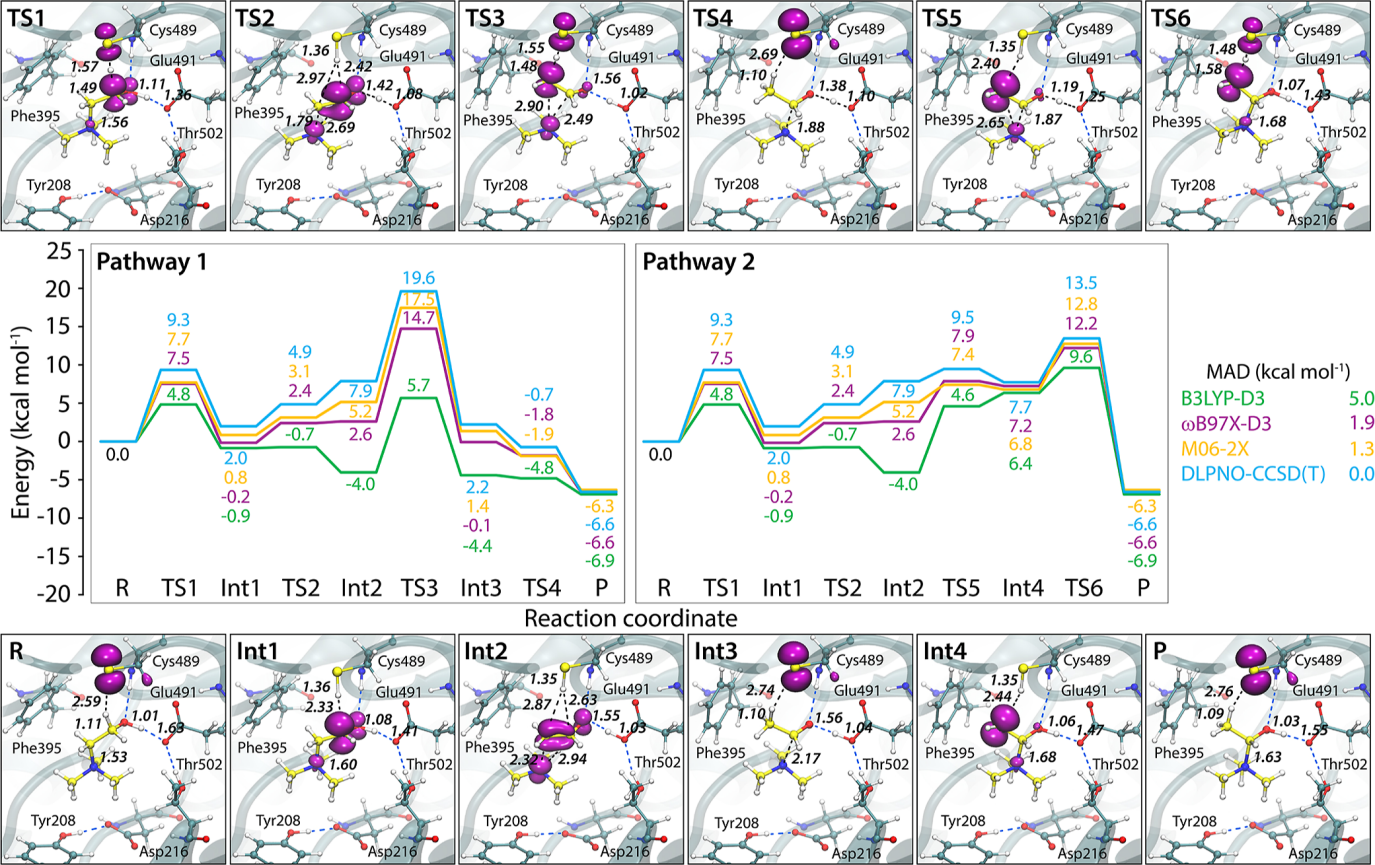
Putative choline cleavage pathways catalyzed by the CutC. Energy profiles were computed using QM/MM at the B3LYP-D3/6-31G­(d) geometry-optimized level, with single-point energies evaluated using B3LYP-D3, ωB97X-D3, and M06-2X functionals with the def2-TZVP basis set. High-level ab initio DLPNO–CCSD­(T)/cc-pVQZ/C single-point QM/MM calculations are used as a reference and to test DFT/MM results. Zero-point energy corrections were computed at the B3LYP-D3/6-31G­(d) level and applied to all stationary points. Radical localization along the reaction coordinate was monitored via spin density analysis, depicted as magenta isosurfaces (isovalue = 0.008 electrons Å^–3^), to track electronic reorganization during bond cleavage. Bond lengths are given in angstrom. See Table S1 for complete relative energy data for all methods.

Both reaction pathways begin with a *pro-S* HAT from the C1 carbon of choline to the thiyl radical (via TS1, Figure 4), accompanied by a contraction of the S_Cys_–Hα bond length from 2.6 Å to 1.4 Å. Simultaneously, the Cα–Hα bond lengthens from 1.1 Å to 2.3 Å, indicating bond cleavage and enabling subsequent deprotonation of the resulting α-hydroxy radical intermediate by Glu491. This deprotonation promotes cleavage of the Cβ–N bond, which increases from 1.5 Å in the R state to 2.3 Å in Int2, yielding a vinoxy radical and TMA.

The pathways diverge here. In pathway 1, a direct HAT occurs from Cys489 to the C2 position of the vinoxy radical (via TS3), as shown by a pronounced elongation of the S_Cys_–Hα bond from 1.4 Å to 2.7 Å, accompanied by a simultaneous shortening of the Cβ–Hα bond from 2.9 Å to 1.1 Å. In pathway 2, a 1,2-migration of the TMA group occurs prior to HAT; this migration is indicated by a decrease in the Cα–N distance from 2.9 Å to 1.7 Å (via TS5). Following migration, HAT from Cys489 to the carbinolamine radical takes place (via TS6), marked by an increase of the S_Cys_–Hα bond length from 1.4 Å to 2.8 Å and a decrease of the Cβ–Hα bond from 2.4 Å to 1.1 Å, reflecting cleavage and formation of S–H and C–H bonds, respectively.

Both pathways conclude with a spontaneous proton transfer from Glu491, producing a chiral carbinolamine and regenerating the catalytically active enzyme. The overall reaction is exergonic with a computed reaction energy of −6.6 kcal mol^–1^. Along the reaction coordinate, the distance between the positively charged TMA nitrogen and the carboxylate oxygen of Glu491 decreases from 4.3 Å to 3.9 Å, implying increased electrostatic stabilization of the carbinolamine relative to choline. Additionally, a notable conformational change in Phe395 is observed, with its Cα–Cβ–Cγ–Cδ dihedral angle increasing from 104° to 122°, to accommodate carbinolamine. These key geometric parameters are summarized in Table S2.

From a computational perspective, the M06-2X and ωB97X-D3 functionals outperform B3LYP-D3 in comparison to the reference DLPNO–CCSD­(T) calculations (Figure 4). B3LYP-D3 underestimates the activation barriers and overestimates intermediate stabilities here, particularly for species with significant spin delocalization, where dynamic correlation effects are pronounced. These findings align with the limitations and strengths of different DFT methods for modeling organic radical reactions.^65,66^

To assess the impact of QM region size on reaction energetics, we performed QM/MM calculations with four progressively larger QM regions (minimal, medium, large, and extra-large). Expanding from the minimal to medium region substantially improved B3LYP-D3 single-point energies, better aligning with high-level DLPNO–CCSD­(T) reference values. Further growth to large and extra-large regions had negligible effect, indicating energy convergence with respect to QM size. Notably, using the M06-2X functional in combination with the extra-large QM region produced the closest agreement to DLPNO–CCSD­(T), underscoring the importance of both functional choice and QM region completeness (see Figure S5).

Importantly, increase of the QM region size beyond the core catalytic residues (Cys489 and Glu491) significantly increased the relative energies of reaction intermediates with B3LYP-D3, a trend also seen with ωB97X-D3 and M06-2X. This effect is attributed to improved treatment of electronic polarization: inclusion of residues forming hydrogen bonds to Glu491 and the choline substrate (Asp216, Thr502) enhanced agreement across functionals, whereas aromatic residues (Tyr208, Phe395) had little impact. These results highlight the necessity of capturing mutual polarization between the substrate and active site, an effect not adequately described by conventional electrostatic embedding approaches.

To evaluate the roles of aromatic Tyr208 and Tyr506 in the reaction mechanism, we conducted a series of QM/MM single-point energy calculations on our optimized geometries, systematically removing the partial charges of these tyrosine residues in the MM region. We first excluded the partial charges of each tyrosine individually, and then both simultaneously. Remarkably, these modifications resulted in only minimal changes to the potential energy profiles compared to calculations retaining the full point-charge representation. This strongly suggests that the electrostatic contributions of Tyr208 and Tyr506 have little direct impact on the 1,2-migration mechanism (see Figure S6).

Complementary MM MD simulations of the Y208F/Y506F double mutant provided important additional insights. In the mutant, we observed the appearance of a new water molecule forming stabilizing hydrogen bonds with Asp216 and the backbone of Thr334, in agreement with observations from the crystal structure of the single Y208F mutant (PDB ID 5FAY).^15^ Most notably, analysis of the free energy landscape revealed increased choline mobility in the active site of the mutant, evidenced by a shift in the probability distribution for key interactions—particularly the S–H contact between choline and Cys489—toward nonreactive conformations (Figure S7).

Taken together, these findings indicate that while the direct electrostatic effects of Tyr208 and Tyr506 are minor, their influence on the surrounding hydrogen-bonding network induces subtle structural rearrangements. These rearrangements increase substrate flexibility and promote nonproductive choline conformations, providing a plausible molecular explanation for the experimentally observed ∼83-fold reduction in catalytic efficiency in the Y208F/Y506F double mutant.

### Proton Transfer and C–N Bond Cleavage in CutC are Coupled, as Revealed by QM/MM MD Simulations

To examine the interplay between deprotonation of the hydroxyl group and C–N bond scission, unbiased B3LYP/MM MD simulations were conducted on CutC complexed with three ligands: choline, the α-hydroxy radical, and carbinolamine. The initial geometries for the α-hydroxy radical and carbinolamine intermediates were generated from QM/MM umbrella sampling MD simulations, which modeled HAT from choline to a thiyl radical and from cysteine to a vinoxy radical, respectively (see Figure S8 for details). The computed PMFs along each hydrogen atom transfer reaction coordinate show good agreement with the corresponding potential energy profiles calculated at a similar level of theory (Figure 4) and are presented in Figure S9. While these umbrella sampling PMFs provide valuable energetic landscapes of the HAT processes, their primary role in this study was to generate reliable starting structures for subsequent, extensive unbiased QM/MM MD simulations. For each system, FELs were constructed using two reaction coordinates: the proton transfer coordinate (describing whether the proton is associated with the ligand or Glu491) and the C–N bond length (indicating the extent of bond cleavage) as shown in Figure 5.

**Figure 5:**
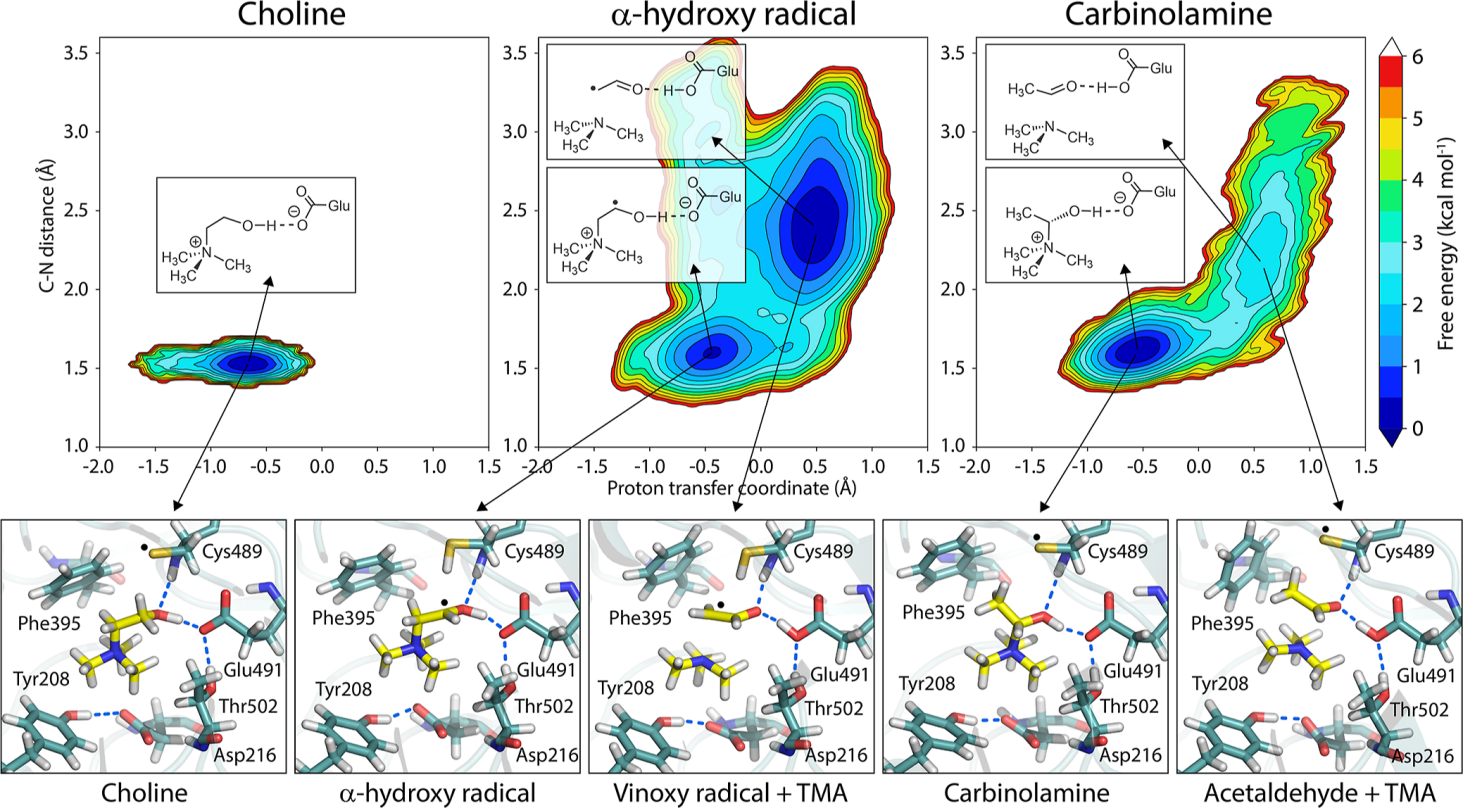
Free energy landscape (FEL) constructed from snapshots of a 50 ps QM/MM molecular dynamics simulation (QM level: B3LYP-D3BJ/6-31G­(d)) of CutC with choline, an α-hydroxy radical, and a carbinolamine intermediate. The FEL is mapped using two reaction coordinates: a proton transfer coordinate, defined as a linear combination of the OH–HO and HO–OE2 (Glu491) bond distances, and the C–N bond distance. The FEL is evaluated at 300 K. See Figure S10 for the time evolution of these distances.

These QM/MM MD simulations demonstrate that, in the choline-bound state, proton transfer does not occur spontaneously, and the C–N bond remains intact (∼1.5 Å) throughout the simulation. In contrast, following HAT from choline to the thiyl radical, the α-hydroxy radical undergoes rapid deprotonation to Glu491, which is immediately followed by C–N bond cleavage to yield a vinoxy radical and trimethylamine (TMA). This process is accompanied by increased flexibility in the active site (Figure 5), suggesting a dynamic environment that facilitates the reaction. The presence of an unpaired electron in the α-hydroxy intermediate appears to promote deprotonation by Glu491, which is tightly correlated with C–N bond cleavage (see Figure S10).

Importantly, although proton transfer and C–N bond cleavage are also observed in the carbinolamine intermediate, leading to the formation of acetaldehyde and TMA, this reaction occurs much less frequently (Figure S10). This reduced reactivity is attributed to the strong electrostatic stabilization of the cationic carbinolamine intermediate by the negatively charged Asp216 and Glu491 residues.

### RBFE Simulations Reveal the Molecular Basis for the Preferential Binding of Carbinolamine to CutC

Elucidating the differences in the interactions of choline and carbinolamine with CutC is critical for rational design of potent enzyme inhibitors. To investigate these differences, the relative binding affinities of choline and carbinolamine were computed using alchemical free energy calculations, performing both the forward transformation (choline to carbinolamine) and the backward transformation (carbinolamine to choline). The resulting ΔΔ*G*_RBFE_ values are summarized in Table 1.

**Table 1:** Relative Binding Free Energies (ΔΔ*G*_RBFE_) Calculated for the Forward (CHT → CBA) and Backward (CBA → CHT) Transformations in Both Radical and Non-Radical Systems^a^

Cys489	transformation	ΔΔ*G*_RBFE_ (kcal mol^–1^)
radical	CHT → CBA	–1.4 ± 0.4
	CBA → CHT	1.9 ± 0.4
nonradical	CHT → CBA	0.3 ± 0.6
	CBA → CHT	1.1 ± 0.4

a Results reflect the differences in binding affinities between the two states. Refer to Figures S11–S18 for the overlap matrices and time convergence analysis.

The computed free energy differences indicate that carbinolamine forms a more stable complex with the active (thiyl radical) form of CutC, compared to choline. In contrast, when CutC is in the nonradical (cysteine) state, the preference for carbinolamine binding is less pronounced. Both ligands form strong hydrogen bonds with the carboxylate group of Glu491, contributing to binding (Figure 6).

**Figure 6:**
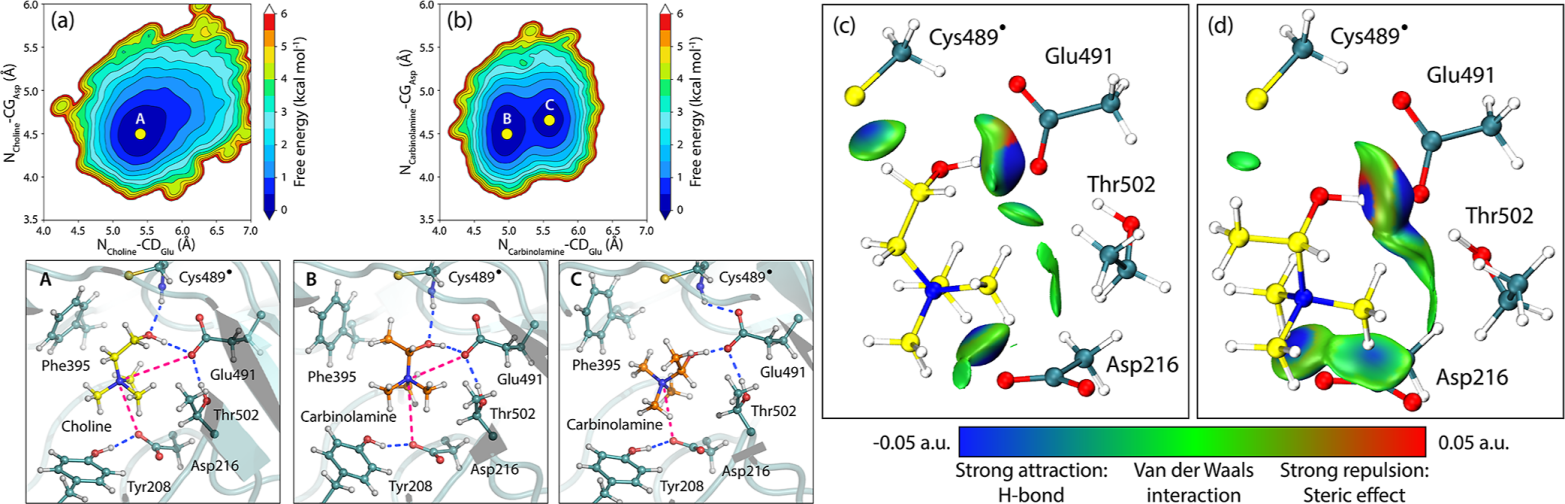
Free energy landscapes (FEL) from MM MD simulations of CutC with (a) choline and (b) carbinolamine, showing dominant substrate conformations and key hydrogen bonds with active site residues. The FELs are constructed using two collective variables: the distances between the positively charged trimethylammonium group and the negatively charged residues Glu491 and Asp216, which are essential for substrate binding and catalysis. The FELs are evaluated at 300 K. Independent gradient model analysis based on *sign*(λ_2_)­ρ from QM/MM MD snapshots (B3LYP-D3BJ/6-31G­(d)) visualizes noncovalent interactions of (c) choline and (d) carbinolamine with the active site as isosurfaces (isovalue 0.01).

Energy decomposition analysis was conducted on the CutC complexes with choline and carbinolamine to elucidate the individual energetic contributions governing ligand binding. The results demonstrate that the total binding free energy (ΔΔ*E* (carbinolamine – Choline) = −1.3 kcal mol^–1^) is primarily dominated by electrostatic (−2.7 kcal mol^–1^) and polar solvation (−0.6 kcal mol^–1^) components, with the carbinolamine complex (Figure S19). Per-residue decomposition identified Glu491 as a major contributor to the electrostatic stabilization, attributable to strong charge–charge interactions with the positively charged nitrogen of carbinolamine positioned in close proximity to the negatively charged side chain of Glu491. Conversely, analysis of van der Waals contributions revealed a more pronounced stabilization in the CutC-choline complex (1.8 kcal mol^–1^), specifically involving Phe395. The van der Waals interaction energy between Phe395 and choline was modestly lower than that with carbinolamine, indicative of enhanced hydrophobic interactions and improved shape complementarity in the choline-bound state.

The difference in ligand contribution to the total binding free energy between carbinolamine and choline was calculated to be −2.3 kcal mol^–1^. This difference is primarily driven by favorable electrostatic (−1.3 kcal mol^–1^) and polar solvation (−1.9 kcal mol^–1^) contributions that enhance carbinolamine binding. Interestingly, the nonpolar solvation term was negligible, while van der Waals interactions favored choline binding by only 0.9 kcal mol^–1^. These ligand-specific energy contributions appear to dominate the overall binding affinity trends observed between the two ligands.

NCI analysis of the electronic structure further explains the differences in binding affinities. Specifically, the TMA group of carbinolamine is positioned to form a stronger stabilizing noncovalent C–H···O interaction with the negatively charged Asp216. Moreover, the TMA cation is closer to Glu491, resulting in additional electrostatic stabilization of the carbinolamine–CutC complex (Figure 6).

## Conclusion

Our integrated simulation study reveals that CutC-catalyzed choline cleavage operates exclusively through a highly selective 1,2-migration mechanism, orchestrated by the interplay of hydrogen bonding, proton transfer, and radical chemistry within the enzyme active site. Extensive MD simulations reveal that the choline substrate is tightly constrained by robust hydrogen bonding and electrostatic interactions into a dominant binding conformation. This preorganization ensures stereoselective *pro-S* hydrogen atom abstraction and effectively suppresses the formation of unstable β-hydroxy radicals.

Critically, our results decisively refute earlier hypotheses involving proton relay and direct TMA elimination.^11,15,17^ These alternative pathways are energetically prohibitive and incompatible with the reaction energetics. QM/MM calculations further establish that the dominant mechanistic pathway, a 1,2-migration of the TMA group, exhibits a calculated activation barrier (Δ*E* ^⧧^_DLPNO–CCSD(T)_ = 13.5 kcal mol^–1^), that closely matches experimental values,^15^ confirming its physiological relevance. The active site residues Asp216 and Glu491 play indispensable roles in stabilizing radical and cationic intermediates as well as transition states.

The QM/MM MD simulations and alchemical free energy calculations reveal the dynamic coupling of proton transfer and C–N bond cleavage, and demonstrate that the carbinolamine intermediate binds more tightly to the enzyme than choline itself. Energy decomposition analysis highlights distinct energetic determinants of ligand specificity in CutC: electrostatic and polar solvation interactions, predominantly mediated by Glu491, are central to carbinolamine binding, whereas hydrophobic van der Waals interactions involving Phe395 are more significant for choline recognition.

From a practical standpoint, these findings provide a robust foundation for the rational design of selective CutC inhibitors. The marked affinity of the carbinolamine intermediate for the active site, especially its close proximity to the charged Asp216 and Glu491 residues, indicates that targeting this region can effectively leverage analogous electrostatic and hydrogen-bonding interactions. Notably, small-molecule halomethylcholines,^5,67^ with carbon–halogen bonds positioned near the enzyme negatively charged residues, stand out as an exceptionally promising scaffold for inhibitor development. The potency of these compounds likely stems from their ability to closely mimic the carbinolamine electronic environment and to foster favorable interactions with key enzyme residues. Moving forward, the integration of electric field calculations and experimental validation will further refine inhibitor optimization, accelerating the discovery of novel agents capable of disrupting microbial choline metabolism.

Collectively, our findings provide molecular-level insight into CutC catalysis, establish a conserved mechanistic paradigm among glycyl radical enzymes, and chart a clear path for the rational design of next-generation inhibitors targeting microbial choline metabolism. These advances not only deepen our understanding of radical enzyme function but also open new avenues for therapeutic intervention in microbiome-associated diseases.

## Supplementary Material



## Data Availability

The QM/MM optimized geometries and AMBER topologies, coordinates, and input files are available on Zenodo 10.5281/zenodo.15386654.

## Notes

Additional simulation details, materials, and methods, including figures, plots, and tables of QM/MM MD and RBFE simulations (PDF)
