# Supplementary material for: All Roads Lead to Carbinolamine: QM/MM Study of Enzymatic C–N Bond Cleavage in Anaerobic Glycyl Radical Enzyme Choline Trimethylamine-Lyase (CutC)
Source: J Phys Chem B. 2025 Sep 8;129(37):9322–32. doi: 10.1021/acs.jpcb.5c04023 (PMC12451663; doi:10.1021/acs.jpcb.5c04023)
Supplement: Supplementary file 1 [file jp5c04023_si_001.pdf]

Supporting Information

All Roads Lead to Carbinolamine: QM/MM Study of Enzymatic C-N Bond Cleavage in Anaerobic Glycyl Radical Enzyme Choline Trimethylamine Lyase (CutC)

Marko Hanzevacki[*,§] J. Jasmin Güven,[†] Philip Hinchliffe,[‡] John Shaw,[‡] Antonia S. J. S. Mey,[†] Natalie Fey,[§] James Spencer[‡] and Adrian J. Mulholland[*,§]

[§]Centre for Computational Chemistry, School of Chemistry, University of Bristol, Bristol BS8 1TS, UK

[†]EaStCHEM School of Chemistry, University of Edinburgh, Edinburgh EH9 3FJ, UK

[‡]School of Cellular and Molecular Medicine, University of Bristol, Bristol BS8 1TD, UK

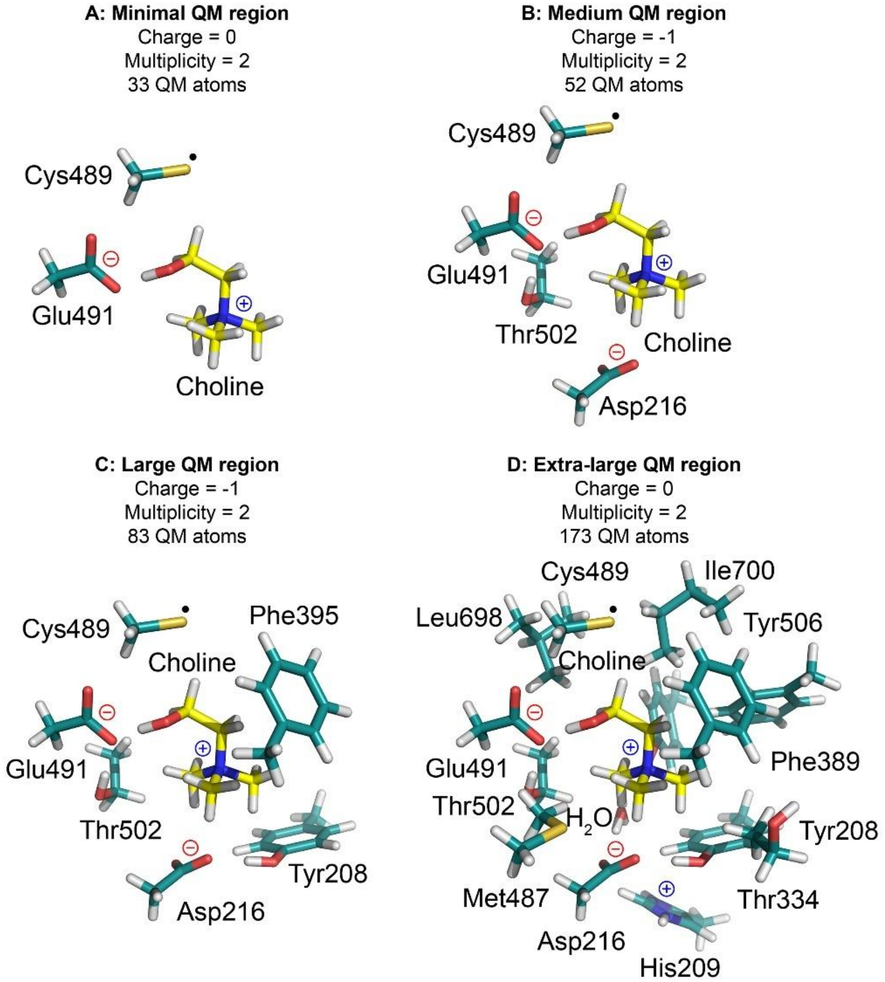

Figure S1 Definition of QM region sizes used in QM/MM calculations. All QM regions were employed for static QM/MM calculations to evaluate their impact on reaction energetics. The medium QM region was used to perform QM/MM molecular dynamics simulations. The QM regions are defined as follows: The minimal QM region includes choline, Cys489, and Glu491. The medium QM region consists of the minimal region plus Asp216 and Thr502. The large QM region further adds Phe395 and Tyr208 to the medium region. The extra-large QM region extends the large region by including an additional water molecule, His209, Thr334, Phe389, Met487, Tyr506, Leu698, and Ile700.

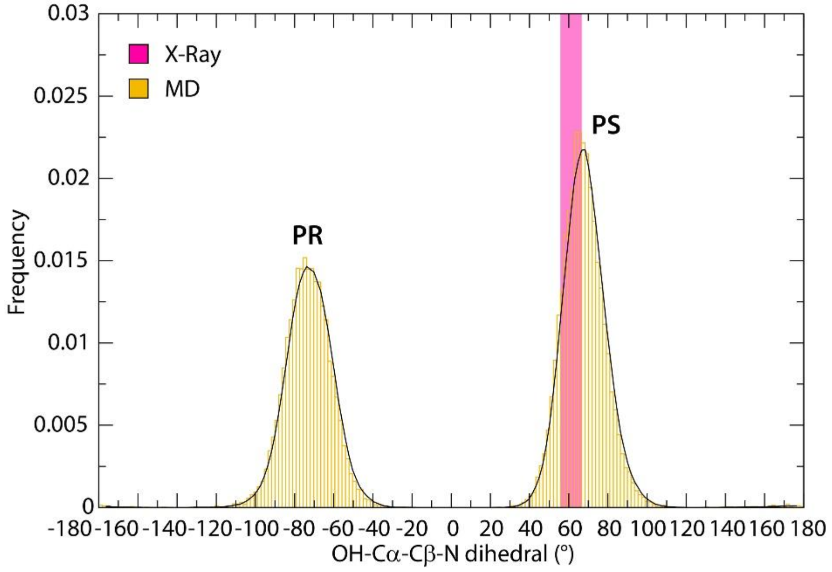

Figure S2 Distribution of conformers obtained by calculating the choline OH-Cα-Cβ-N dihedral from MM MD simulations. Dominant conformation PS closely resembles the conformation of choline in the crystal structure (PDB ID 5FAU).

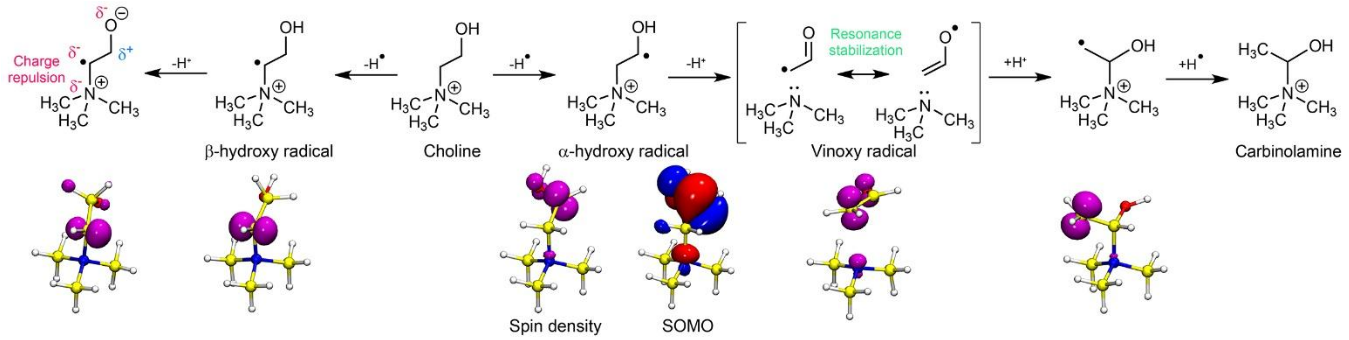

Figure S3 Characterization of possible intermediates following initial hydrogen abstraction and subsequent deprotonation of choline. Spin density distributions (magenta isosurfaces) and singly occupied molecular orbitals (SOMO; blue and red isosurfaces) were calculated at the B3LYP-D3BJ/def2-SVP level of theory, providing insight into the electronic structure of key radical intermediates.

Table S1 Relative QM/MM energies. Mean absolute deviation (MAD) was computed for each functional using a reference DLPNO-CCSD(T) calculations. All values are given in kcal mol^{-1}.

	B3LYP-D3/6-31G(d)		B3LYP-D3/6-311++G(3df,3pd)	B3LYP-D3/def2-TZVP	ωB97X-D3/def2-TZVP	M06-2X/def2-TZVP	DLPNO-CCSD(T)/cc-pVQZ/C
	Δ*E*	Δ*ZPE*			Δ*E*		
R	0.0	0.0	0.0	0.0	0.0	0.0	0.0
TS1	10.4	-5.2	10.2	10.0	12.7	12.9	14.6
Int1	5.8	-3.7	3.0	2.9	3.6	4.6	5.7
TS2	6.7	-5.2	4.9	4.4	7.6	8.3	10.0
Int2	3.9	-5.4	2.2	1.4	8.1	10.6	13.3
TS3	13.8	-7.8	14.2	13.5	22.6	25.3	27.5
Int3	-2.4	-3.5	-0.1	-0.9	3.5	4.9	5.8
TS4	-2.1	-3.9	-0.3	-1.0	2.0	2.0	3.1
TS5	14.7	-7.8	12.8	12.4	15.6	15.2	17.2
Int4	13.9	-4.8	11.2	11.1	12.0	11.6	12.5
TS6	17.5	-6.5	16.2	16.1	18.7	19.2	20.0
P	-5.9	-1.0	-5.8	-5.9	-5.6	-5.3	-5.6
MAD	4.2		4.6	5.0	1.9	1.3	

Table S2 Selected bonds lengths (Å) and Phe395 Cα-Cβ-Cγ-Cδ dihedral angle (°) along the reaction coordinate obtained with QM/MM (QM level: B3LYP-D3/6-31G(d)) calculations.

	Pathway 1									
	Bond									Dihedral
	S_{Cys}-Hα	Cα-Hα	Cβ-Hα	Cβ-N	Cα-N	OH-HO	O_{Glu}-HO	N-O_{Asp}	N-O_{Glu}	Phe395
R	2.59	1.11	2.10	1.53	2.59	1.01	1.63	3.73	4.33	104.23
TS1	1.57	1.49	2.32	1.56	2.58	1.11	1.36	4.02	4.53	104.98
Int1	1.36	2.33	3.00	1.60	2.58	1.08	1.41	4.03	4.74	105.29
TS2	1.36	2.42	2.97	1.79	2.69	1.42	1.08	4.15	4.90	106.85
Int2	1.35	2.63	2.87	2.32	2.94	1.56	1.03	4.25	5.14	109.71
TS3	1.55	2.26	1.48	2.90	2.49	1.56	1.03	4.32	4.54	121.10
Int3	2.73	2.12	1.10	2.82	2.17	1.56	1.04	4.18	4.20	123.13
TS4	2.69	2.12	1.10	2.69	1.88	1.38	1.10	4.02	4.03	122.73
P	2.76	2.12	1.09	2.58	1.63	1.03	1.55	3.76	3.90	122.10

	Pathway 2									
	Bond									Dihedral
	S_{Cys}-Hα	Cα-Hα	Cβ-Hα	Cβ-N	Cα-N	OH-HO	O_{Glu}-HO	N-O_{Asp}	N-O_{Glu}	Phe395
R	2.59	1.11	2.10	1.53	2.59	1.01	1.63	3.73	4.33	104.23
TS1	1.57	1.49	2.32	1.56	2.58	1.11	1.36	4.02	4.53	104.98
Int1	1.36	2.33	3.00	1.60	2.58	1.08	1.41	4.03	4.74	105.29
TS2	1.36	2.42	2.97	1.79	2.69	1.42	1.08	4.15	4.90	106.85
Int2	1.35	2.63	2.87	2.32	2.94	1.56	1.03	4.25	5.14	109.71
TS5	1.35	2.84	2.40	2.65	1.87	1.19	1.25	3.89	3.94	122.28
Int4	1.35	2.90	2.44	2.57	1.68	1.06	1.47	3.86	3.87	122.36
TS6	1.48	2.45	1.58	2.59	1.68	1.07	1.43	4.16	3.88	124.57
P	2.76	2.12	1.09	2.58	1.63	1.03	1.55	3.76	3.90	122.10

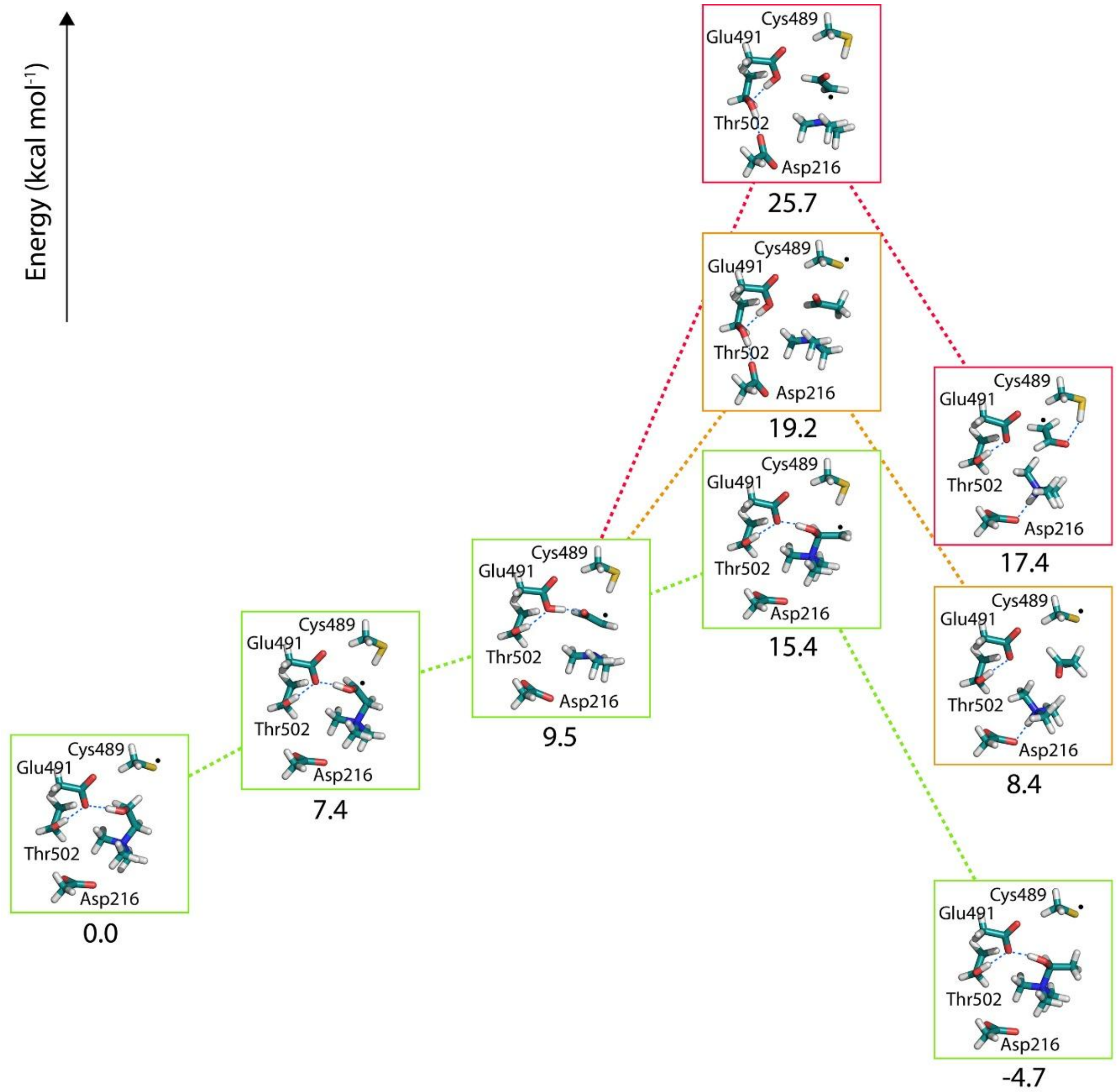

Figure S4 Relative QM/MM energies (QM level: B3LYP-D3/6-31G(d)) and the corresponding structures comparing the two possible choline cleavage pathways: TMA migration (green) and direct TMA elimination (orange and red). Only QM region is shown and the rest of the protein is omitted for clarity.

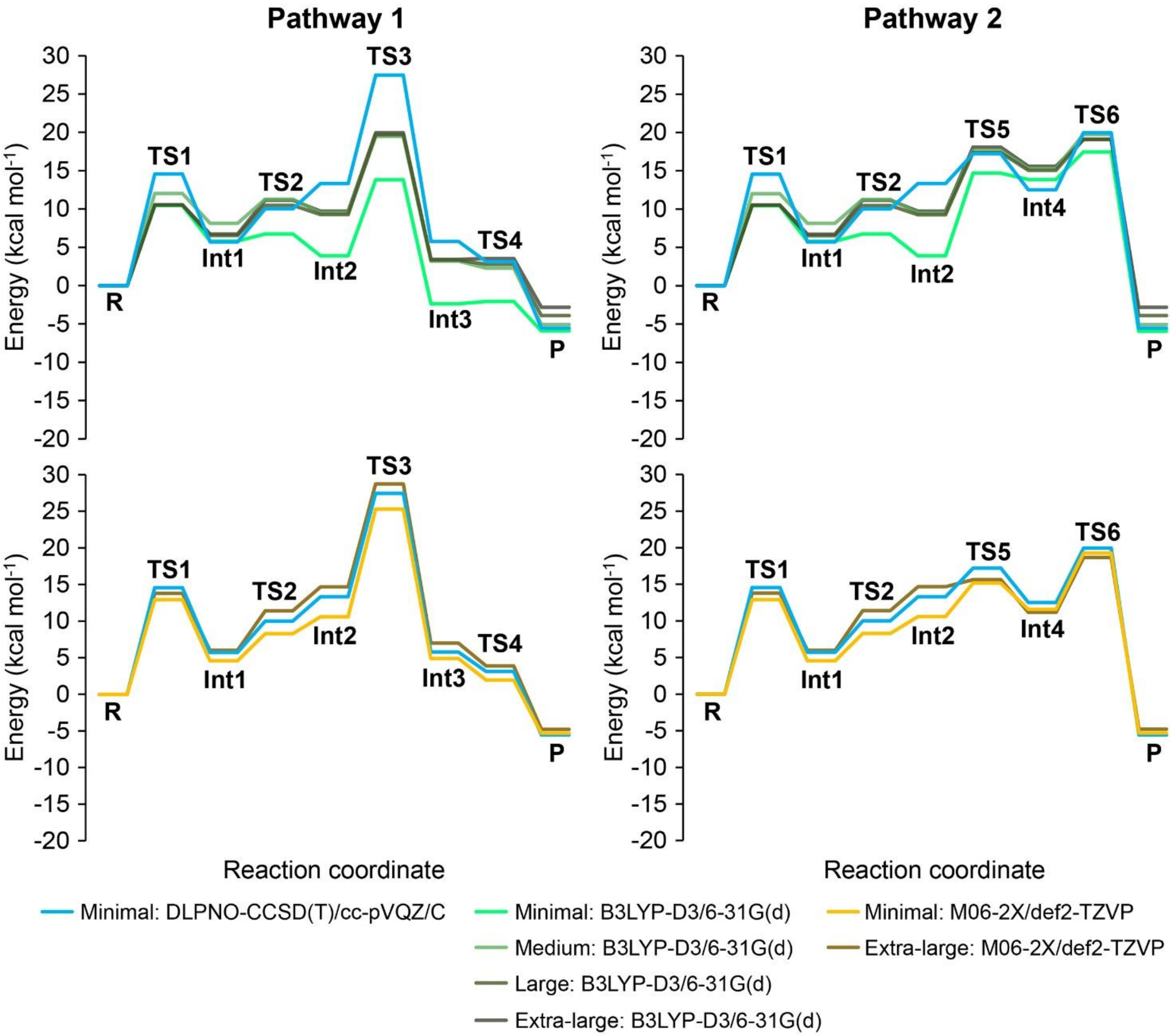

Figure S5 Potential energy profiles for choline cleavage by CutC computed with QM/MM single-point energy calculations using varying QM region sizes. All geometries were optimized at the B3LYP-D3/6-31G(d) level with the minimal QM region. Single-point energies were calculated using B3LYP-D3/6-31G(d) and M06-2X/def2-TZVP. High-level *ab initio* DLPNO-CCSD(T)/cc-pVQZ/C QM/MM single-point calculations serve as the reference. Zero-point energy corrections were not included in these energy profiles.

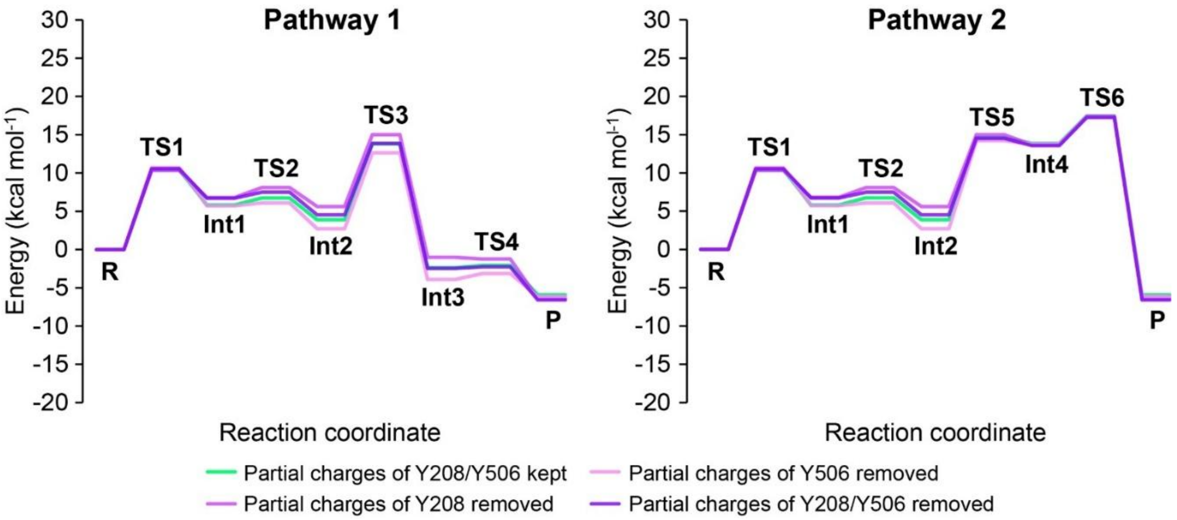

Figure S6 Potential energy profiles for choline cleavage by CutC computed with QM/MM single-point energy calculations with exclusion of point charges from Tyr208, Tyr506, and both Tyr208/Tyr506. All geometries were optimized at the B3LYP-D3/6-31G(d) level using the minimal QM region. Single-point energies were evaluated at the B3LYP-D3/6-31G(d) level without zero-point energy corrections.

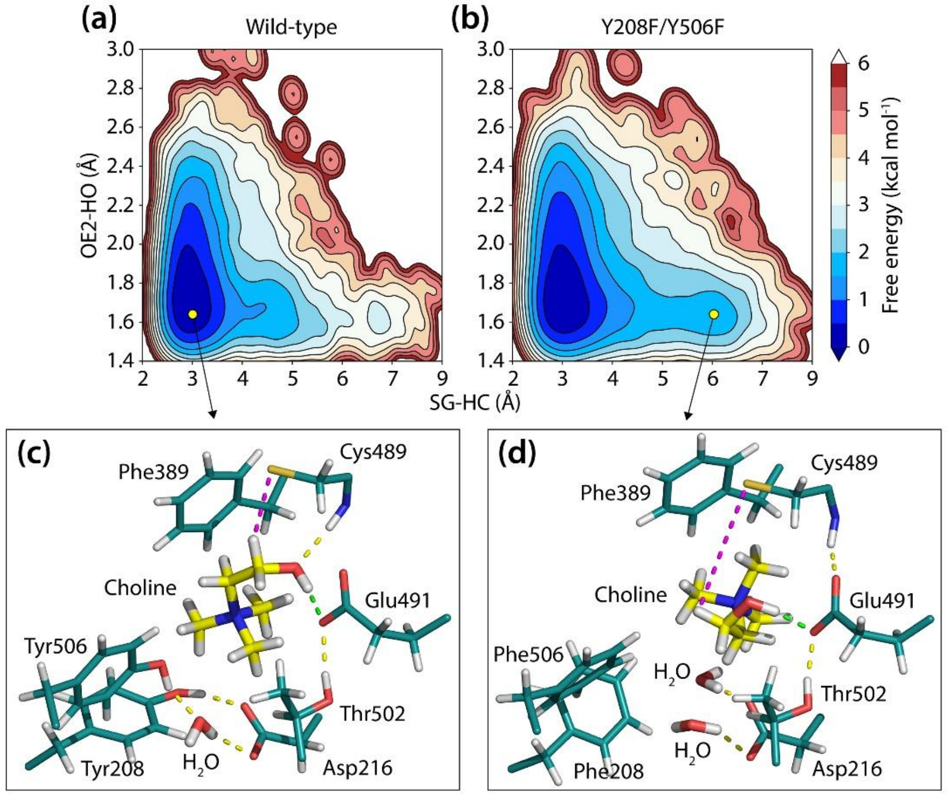

Figure S7 Free energy landscape (FEL) of choline binding in (a) wild-type and (b) Y208F/Y506F double mutant CutC derived from MM MD simulations at 300 K. Representative snapshots illustrate the dominant reactive choline conformation observed in the wild-type active site (c) and the increased choline flexibility favoring non-reactive conformations in the double mutant (d). For clarity, only key residues are shown. The FEL was constructed using two collective variables: the distance between the *pro-S* hydrogen of choline and the sulfur atom of Cys489 (magenta dashed lines), and the distance between the choline hydroxy proton and the OE2 atom of Glu491 (green dashed lines).

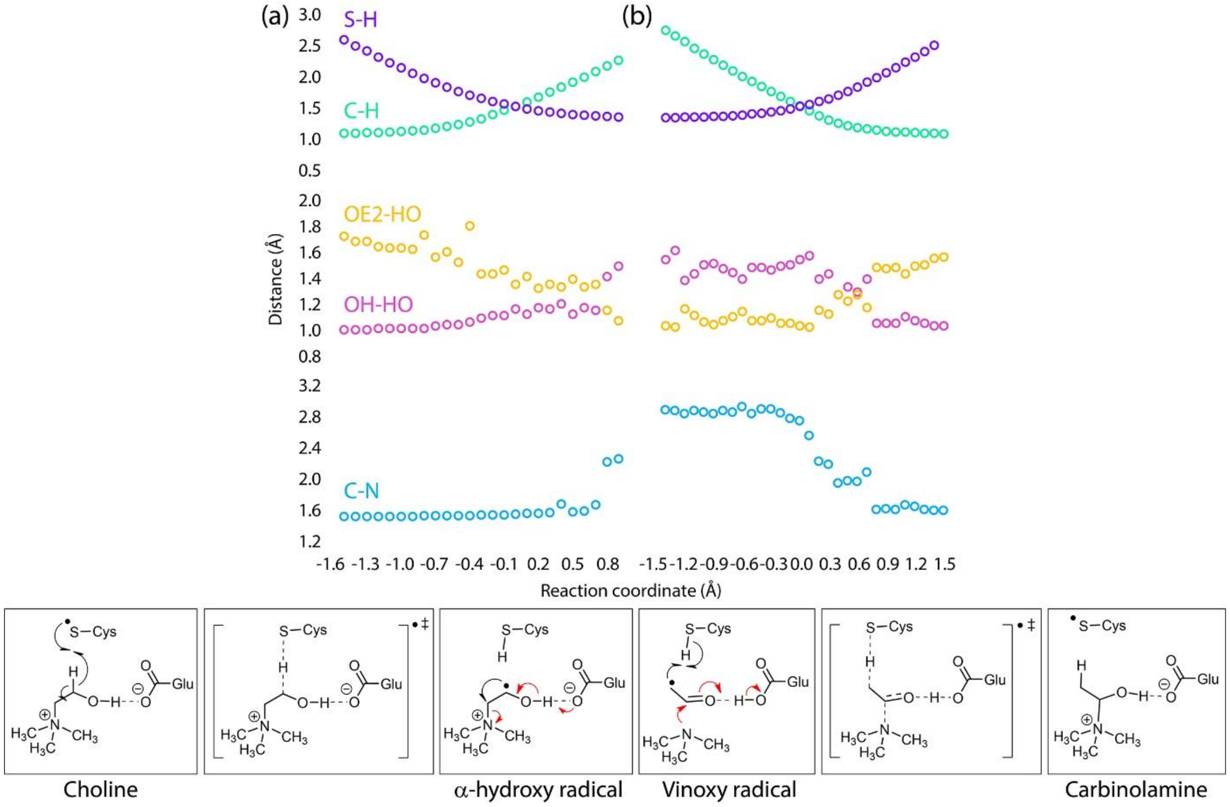

Figure S8 Umbrella sampling QM/MM molecular dynamics simulations (QM level: B3LYP-D3BJ/6-31G(d)) investigating hydrogen atom transfer reactions: (a) from choline to a thiyl radical, and (b) from cysteine to a vinoxy radical. The reaction coordinate is defined as a linear combination of the S–H (cysteine sulfur–hydrogen) and H–C (hydrogen–substrate carbon) bond distances. These simulations provide insight into the mechanistic pathways and structural changes associated with each hydrogen transfer process.

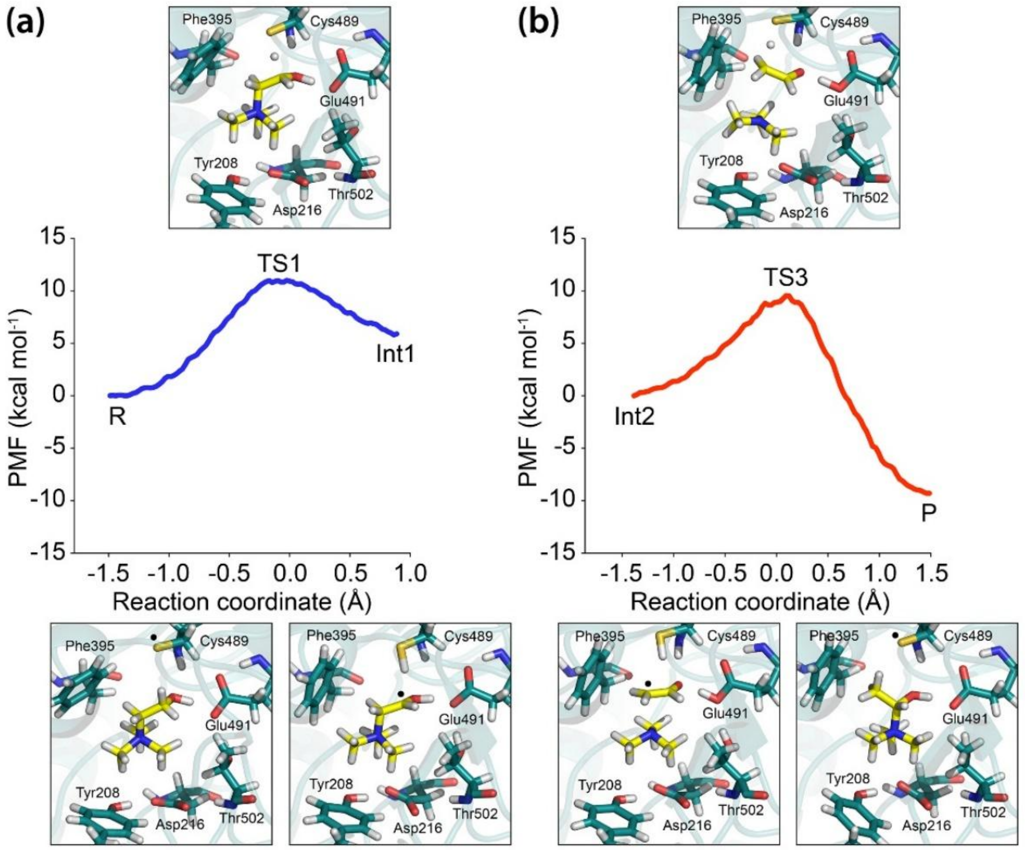

Figure S9 Potential of mean force (PMF) profiles for hydrogen atom transfer reactions: (a) from choline to a thiyl radical, resulting in the formation of an α-hydroxy radical, and (b) from Cys489 to a vinoxy radical, resulting in carbinolamine formation. PMF profiles were calculated using umbrella sampling QM/MM MD simulations at the B3LYP-D3BJ/6-31G(d) level of theory.

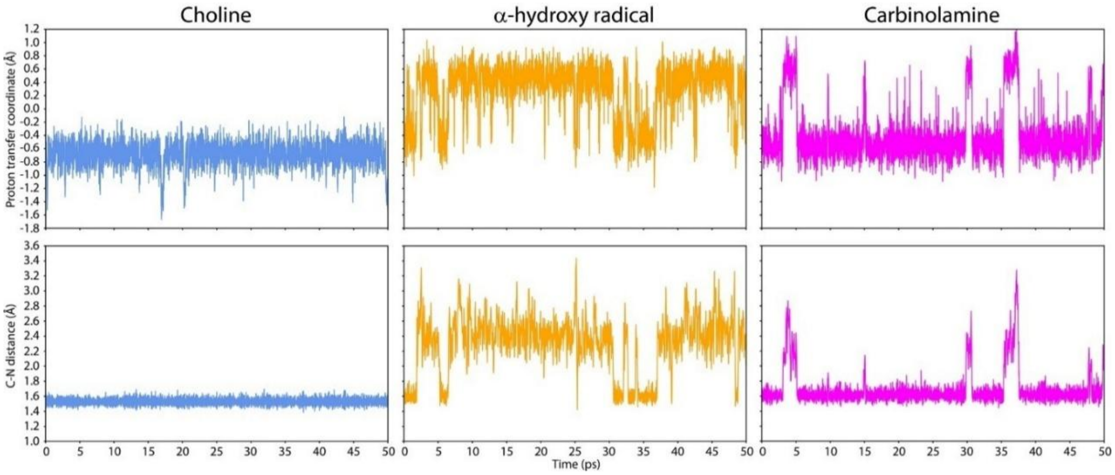

Figure S10 Time evolution of two reaction coordinates during a 50 ps QM/MM molecular dynamics simulation (QM level: B3LYP-D3BJ/6-31G(d)) of CutC with choline, α-hydroxy radical, and carbinolamine. The proton transfer coordinate is defined as a linear combination of the OH–HO (substrate) and HO–OE2 (Glu491) bond distances. The C–N bond distance corresponds to the distance between the substrate's carbon and nitrogen atoms. Distances were calculated throughout the simulation to monitor key chemical transformations within the active site.

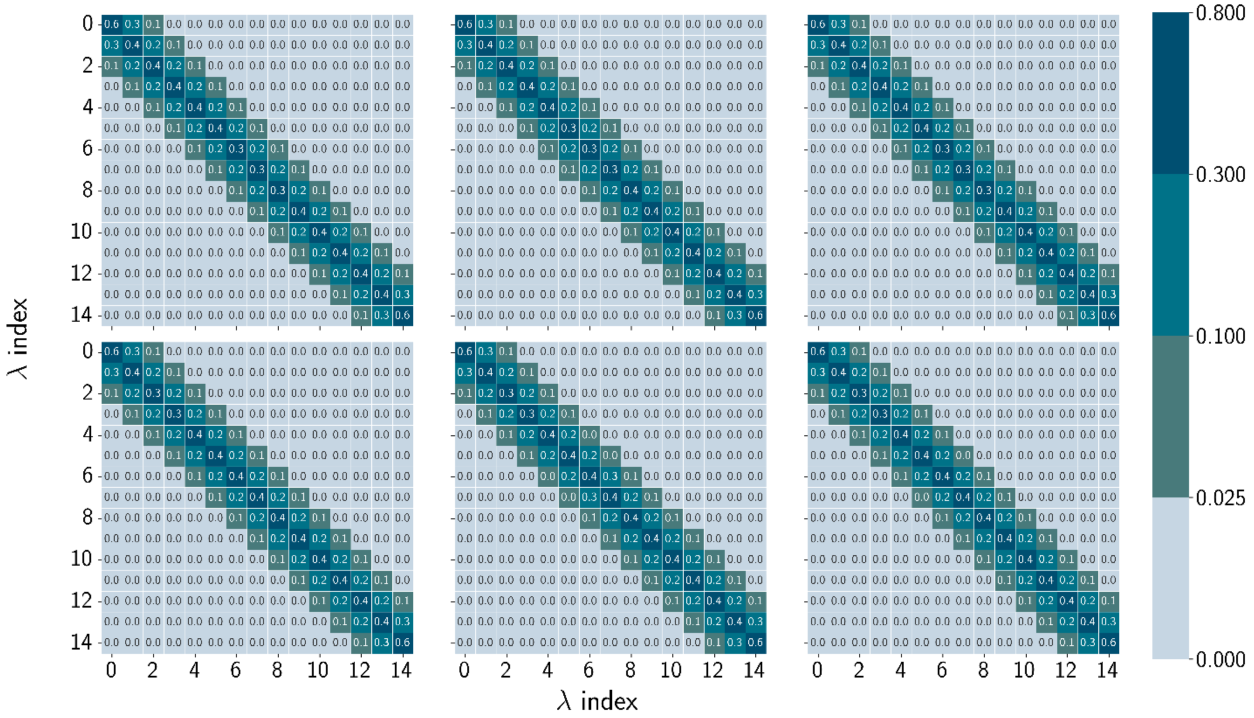

Figure S11 Overlap matrices for the forward transformation of CHT into CBA for the cysteine radical CutC for each repeat in the unbound and bound simulations showing significant overlap between consecutive windows.

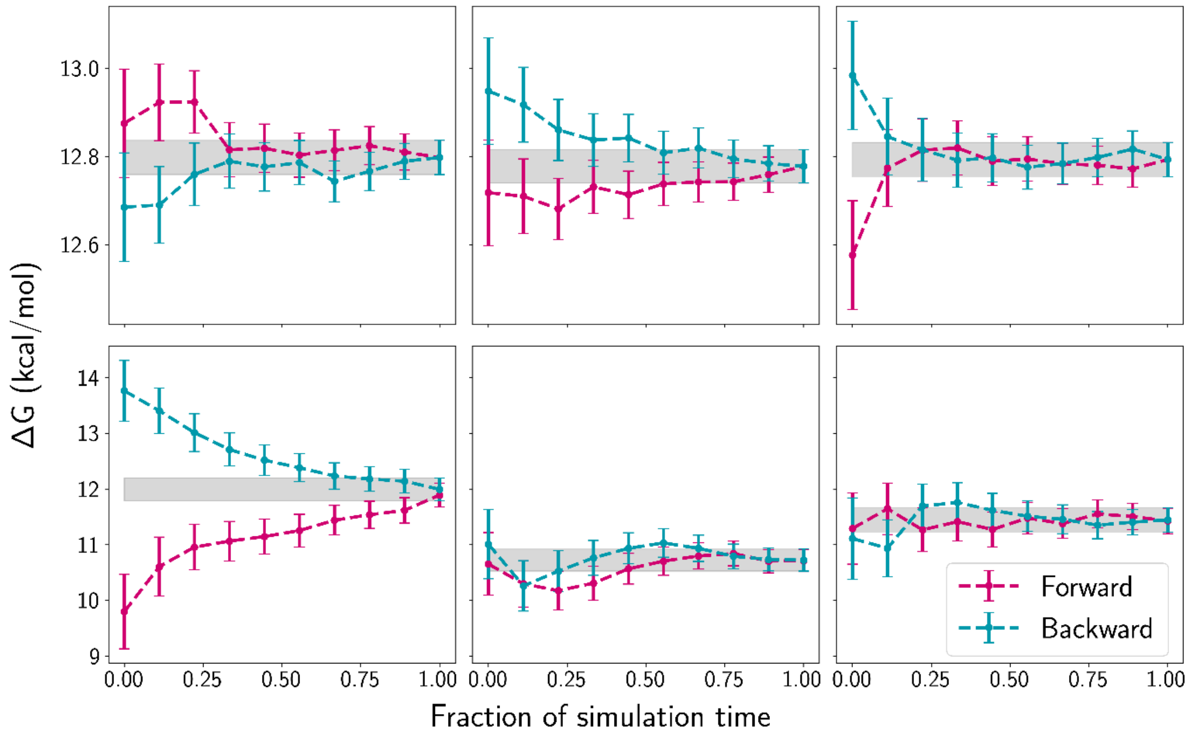

Figure S12 Time convergence for the forward transformation of CHT into CBA for the cysteine radical CutC for each repeat in the unbound and bound simulations showing good convergence. Shaded regions indicated variability in final converged value.

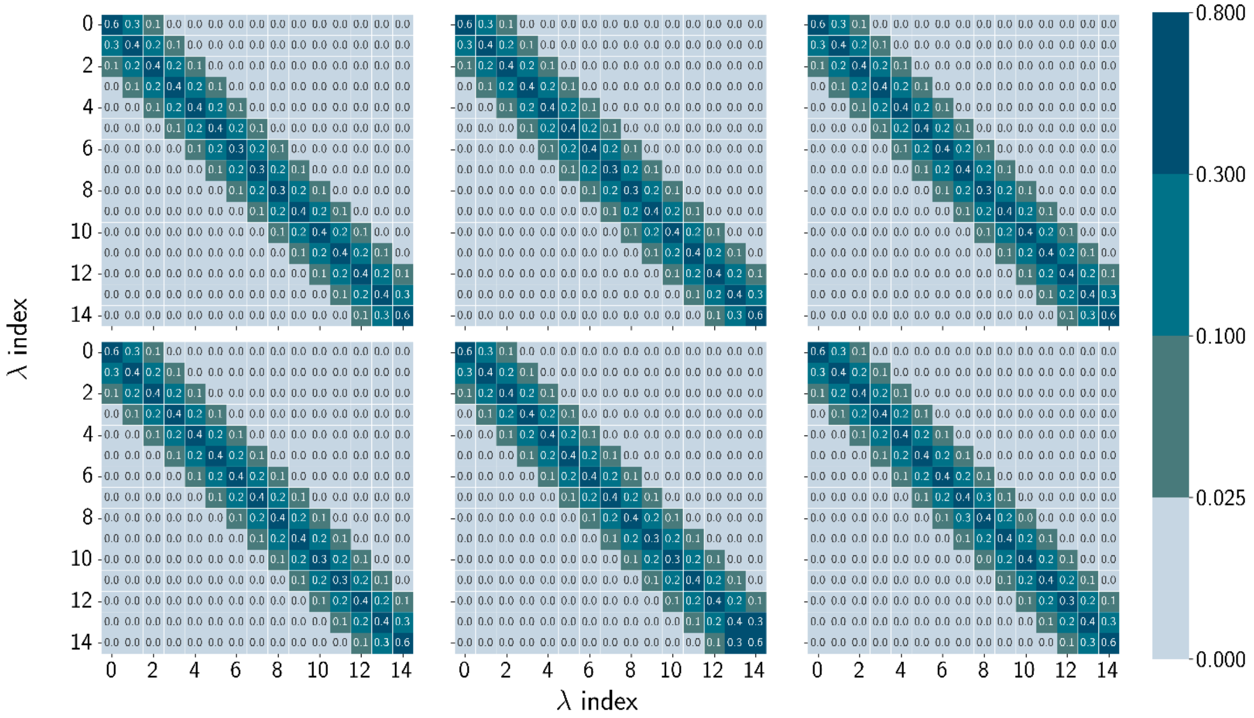

Figure S13 Overlap matrices for the backward transformation of CBA into CHT for the cysteine radical CutC for each repeat in the unbound and bound simulations showing significant overlap between consecutive windows.

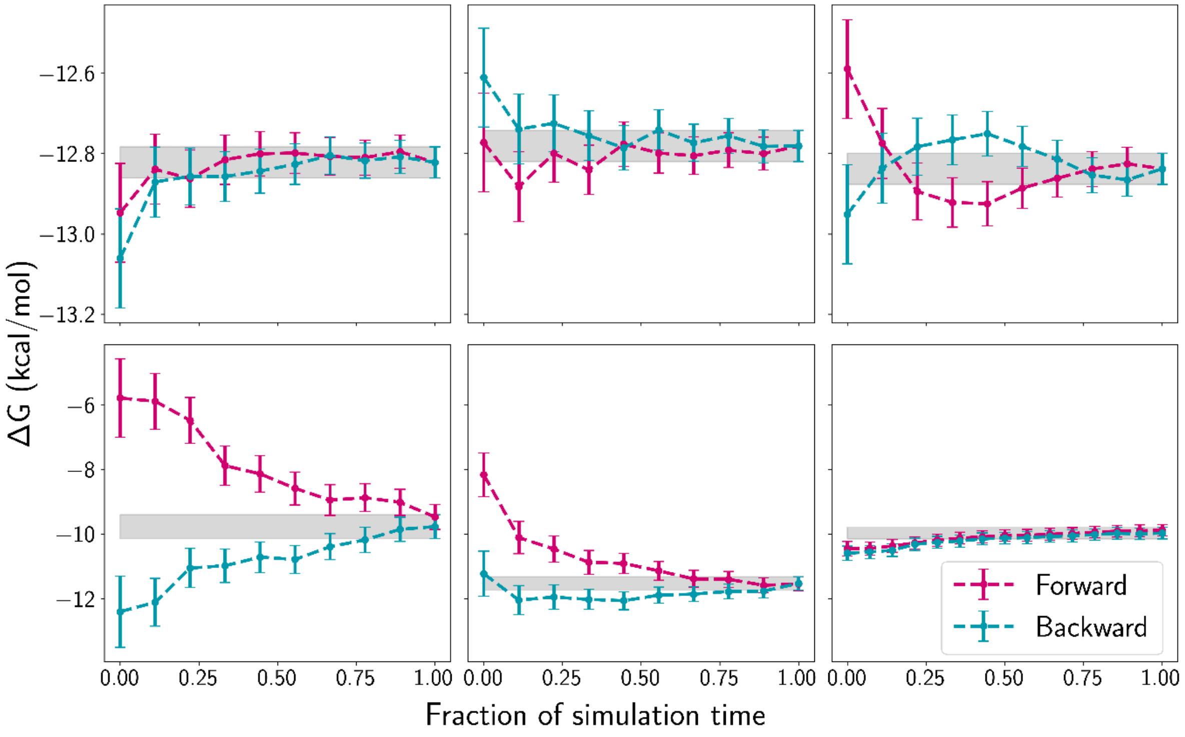

Figure S14 Time convergence for the backward transformation of CBA into CHT for the cysteine radical CutC for each repeat in the unbound and bound simulations showing good convergence. Shaded regions indicated variability in final converged value.

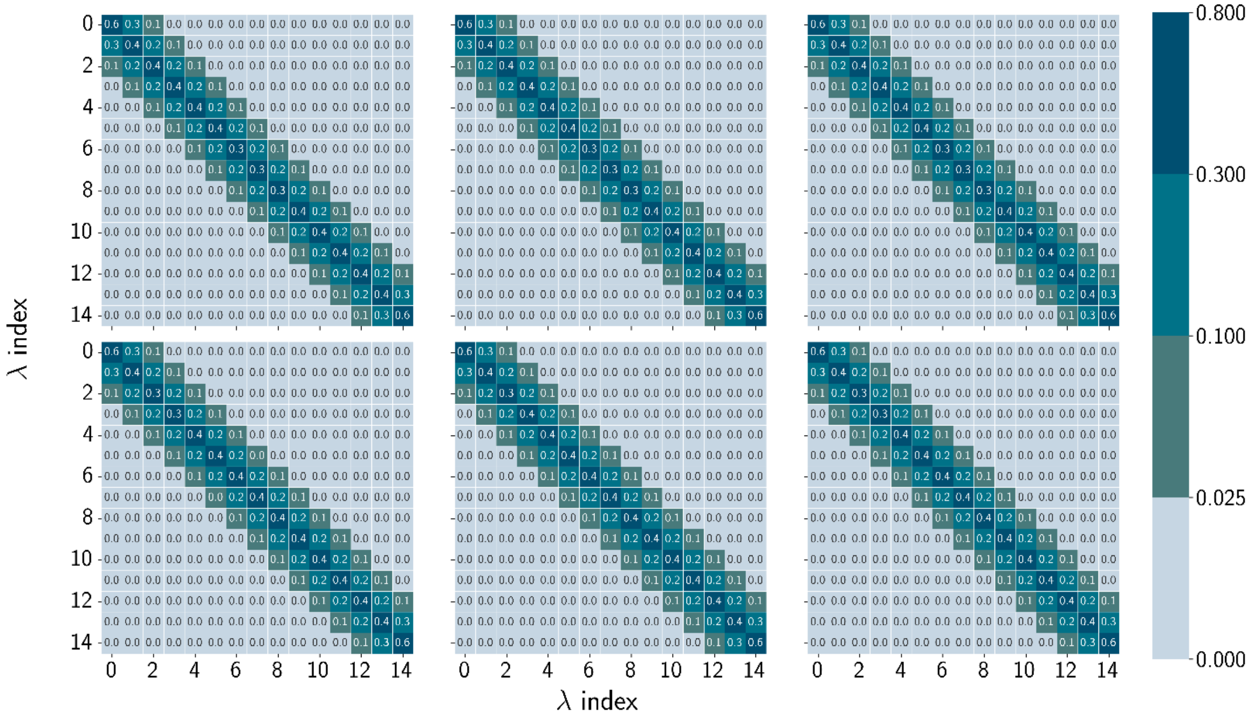

Figure S15 Overlap matrices for the forward transformation of CHT into CBA for the non-radical CutC for each repeat in the unbound and bound simulations showing significant overlap between consecutive windows.

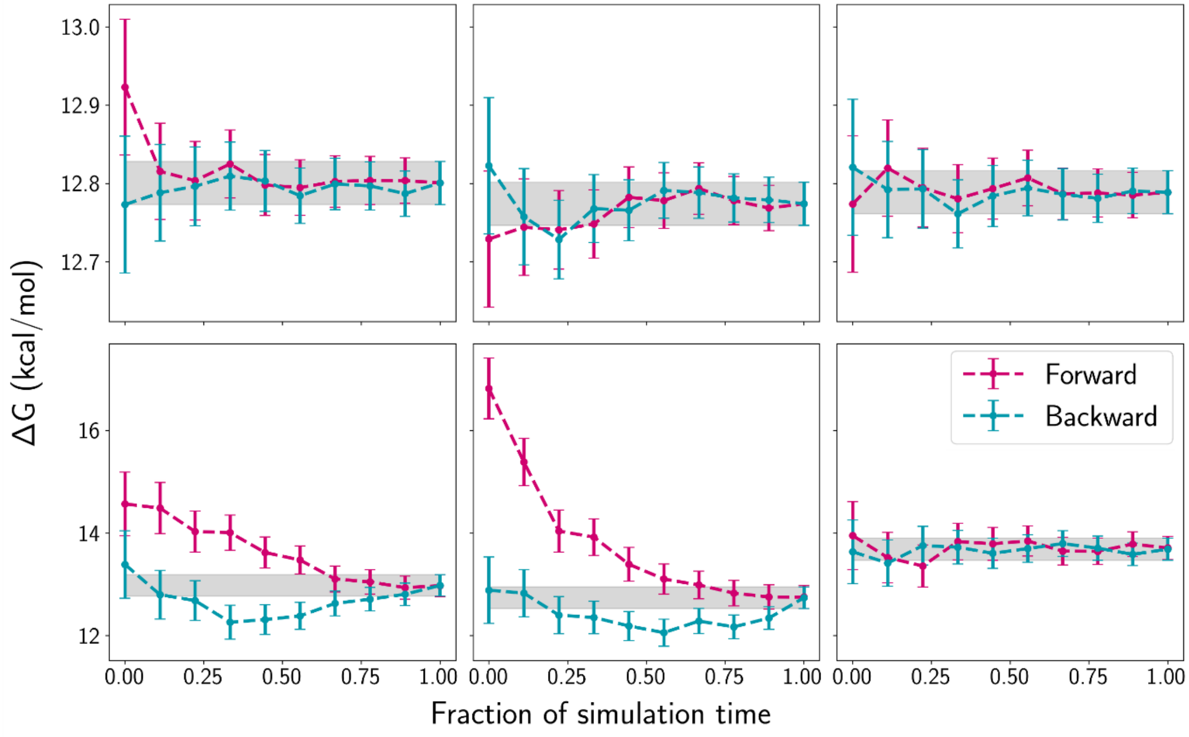

Figure S16 Time convergence for the forward transformation of CHT into CBA for the non-radical CutC for each repeat in the unbound and bound simulations showing good convergence. Shaded regions indicated variability in final converged value.

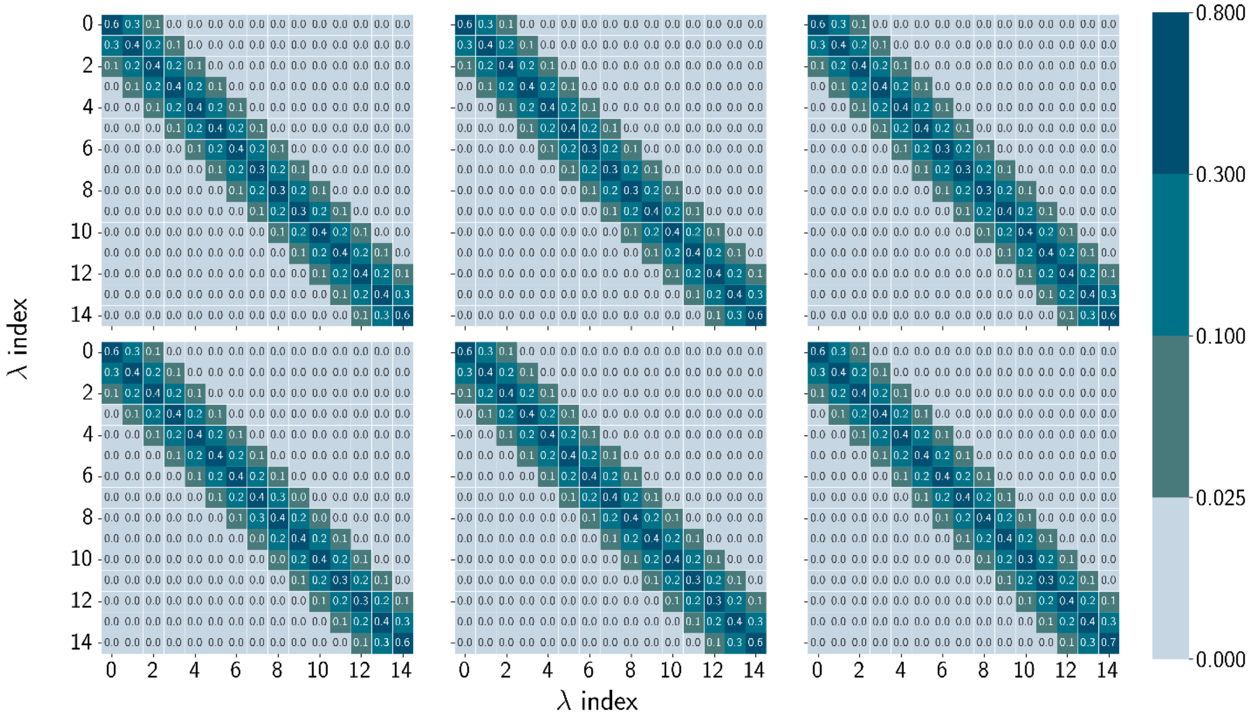

Figure S17 Overlap matrices for the backward transformation of CBA into CHT for the non-radical CutC for each repeat in the unbound and bound simulations showing significant overlap between consecutive windows.

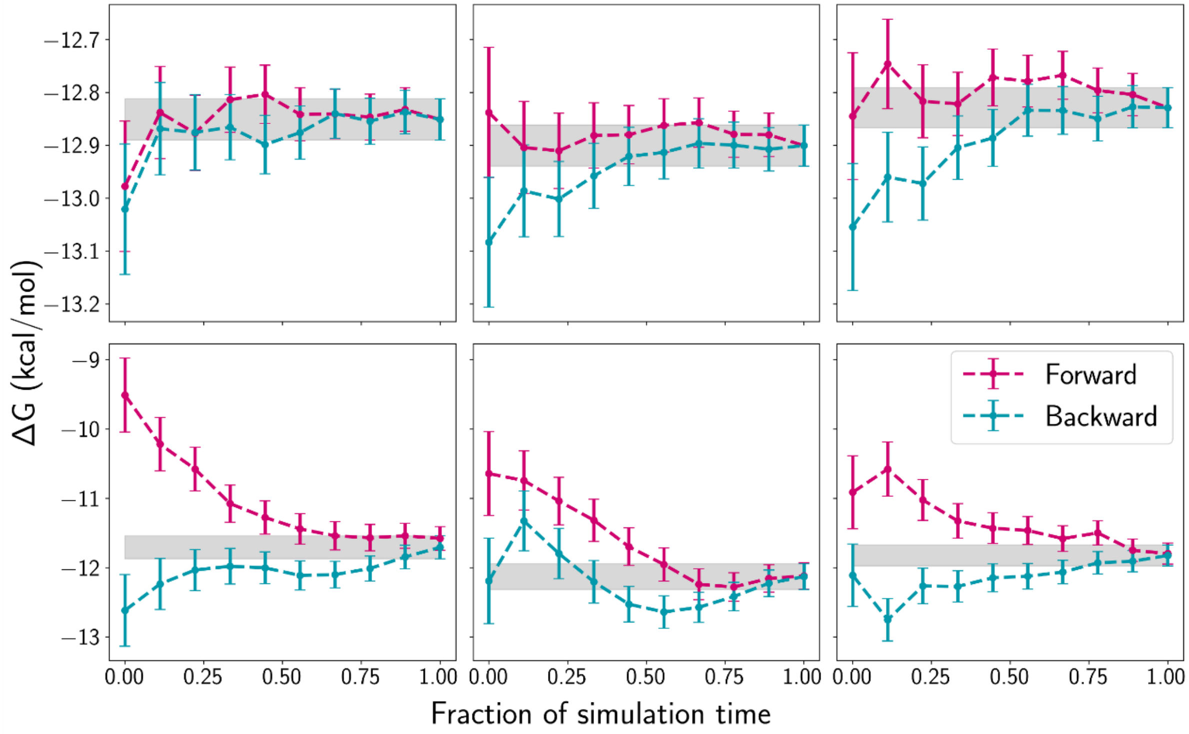

Figure S18 Time convergence for the backward transformation of CBA into CHT for the non-radical CutC for each repeat in the unbound and bound simulations showing good convergence. Shaded regions indicated variability in final converged value.

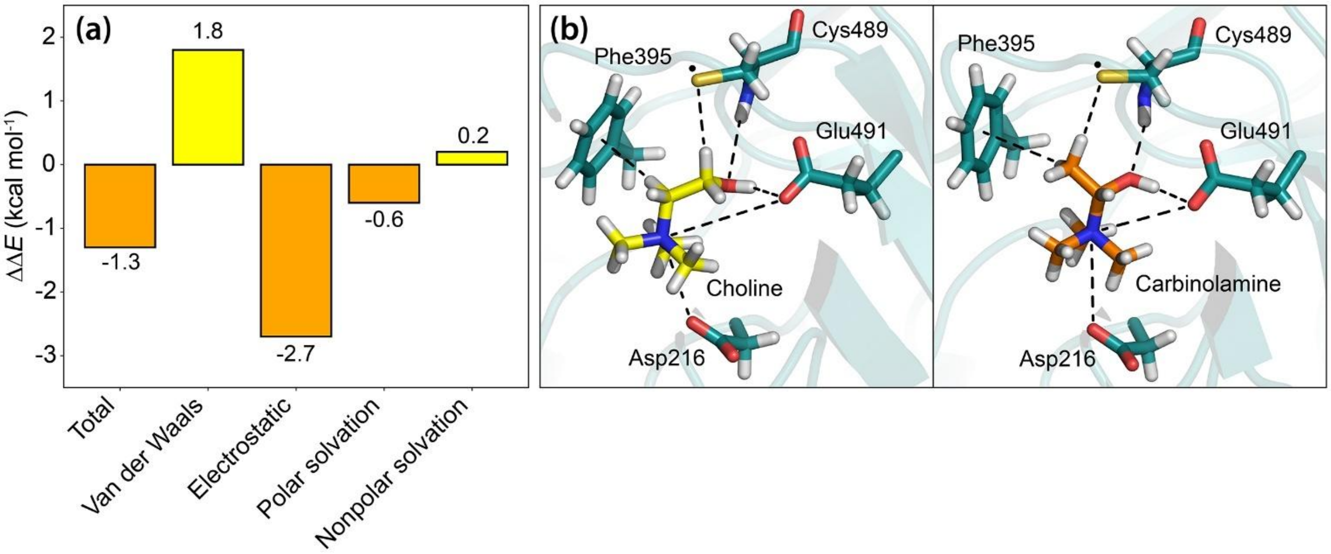

Figure S19 (a) Differences in individual energy components for carbinolamine versus choline binding to CutC. Orange bars indicate energy terms that favor carbinolamine binding, while yellow bars indicate terms more favorable for choline binding. The total binding free energy is the sum of van der Waals, electrostatic, polar solvation (EGB), and nonpolar solvation (ESURF) components. EGB represents the electrostatic contribution to solvation free energy (calculated by the generalized Born (GB) model), and ESURF is the nonpolar contribution determined by the LCPO method. (b) Representative MD simulation snapshots of CutC in complex with choline and carbinolamine.
